# Tyrosine glycosylation of Rho by *Yersinia* toxin impairs blastomere cell behaviour in zebrafish embryos

**DOI:** 10.1038/ncomms8807

**Published:** 2015-07-20

**Authors:** Thomas Jank, Stephanie Eckerle, Marcus Steinemann, Christoph Trillhaase, Marianne Schimpl, Sebastian Wiese, Daan M. F. van Aalten, Wolfgang Driever, Klaus Aktories

**Affiliations:** 1 Institute of Experimental and Clinical Pharmacology and Toxicology, Albert-Ludwigs-University Freiburg, D-79104 Freiburg, Germany; 2 Department of Developmental Biology, Institute Biology I, Faculty of Biology, Albert-Ludwigs-University Freiburg, D-79104 Freiburg, Germany; 3 MRC Protein Phosphorylation and Ubiquitylation Unit, College of Life Sciences, University of Dundee, Dow Street, Dundee DD1 5EH, UK; 4 Center for Biological Systems Analysis, Albert-Ludwigs-University Freiburg, D-79104 Freiburg, Germany; 5 Centre for Biological Signalling Studies (BIOSS), Albert-Ludwigs-University Freiburg, D-79104 Freiburg, Germany

## Abstract

*Yersinia* species cause zoonotic infections, including enterocolitis and plague. Here we studied *Yersinia ruckeri* antifeeding prophage 18 (Afp18), the toxin component of the phage tail-derived protein translocation system Afp, which causes enteric redmouth disease in salmonid fish species. Here we show that microinjection of the glycosyltransferase domain Afp18^G^ into zebrafish embryos blocks cytokinesis, actin-dependent motility and cell blebbing, eventually abrogating gastrulation. In zebrafish ZF4 cells, Afp18^G^ depolymerizes actin stress fibres by mono-*O*-GlcNAcylation of RhoA at tyrosine-34; thereby Afp18^G^ inhibits RhoA activation by guanine nucleotide exchange factors, and blocks RhoA, but not Rac and Cdc42 downstream signalling. The crystal structure of tyrosine-GlcNAcylated RhoA reveals an open conformation of the effector loop distinct from recently described structures of GDP- or GTP-bound RhoA. Unravelling of the molecular mechanism of the toxin component Afp18 as glycosyltransferase opens new perspectives in studies of phage tail-derived protein translocation systems, which are preserved from archaea to human pathogenic prokaryotes.

## Abstract (web summary)

*Yersinia ruckeri* is the source of redmouth disease in fish. Here the authors analysed the *Yersinia* toxin Afp18 and show that it acts to inhibit RhoA activation by glycosylating a distinct tyrosine residue inducing a signalling incompetent structural conformation.

## Graphical abstract

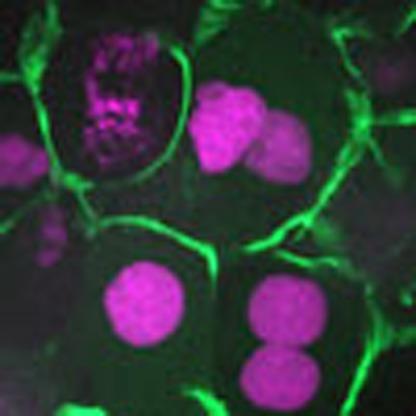

Tight regulation of cytoskeletal organization and activity enables cells to assemble and function in an organism^1^, but the cytoskeleton is also a target of infectious agents causing severe pathologies. Bacterial virulence factors post-translationally modify key regulators of the cytoskeleton, like the family of Rho GTPases, to disseminate or evade host defense mechanisms^2^,^3^,^4^,^5^. The molecular identification of toxins selectively modifying regulatory components of the cytoskeleton can also be useful to uncover cellular processes, equally important for morphogenesis and disease pathogenesis^6^,^7^. Therefore, insights into virulence mechanisms of toxins, particular those modulating Rho GTPases, may promote the understanding of the control of cellular morphology, motility and cell division.

Rho GTPases control cell shape, polarity and motility by regulation of the actin cytoskeleton^1^,^8^,^9^. Accordingly, Rho GTPase signalling regulates germ cell migration in the embryo^10^, and epithelial-to-mesenchymal transition initiating neural crest cell migration^11^. Controlled by planar cell polarity signalling, RhoA and its effectors regulate convergence and extension movements of embryonic tissue during gastrulation^12^,^13^,^14^. Functions of specific Rho GTPases have been analysed during cancer progression^15^, as well as during pathogenic processes on bacterial infection^16^. The use of Rho-specific toxins^17^ and the discovery of pharmacological inhibitors^18^ of Rho signalling were essential to uncover developmental as well as disease-related mechanisms.

Several *Yersinia* species are highly pathogenic for humans and animals causing diseases as diverse as plague and enterocolitis. *Y. ruckeri* is the causative agent of enteric redmouth disease, a generalized septicaemia in salmonid fish species^19^. Despite the economic significance of the disease, very little is known about the pathogenesis and even less is reported on molecular virulence mechanisms. Virulence is presumed to be mediated by a phage-derived needle-like particle complex called antifeeding prophage (Afp)^20^, which shares key characteristics in common with type VI secretion systems (T6SS), R-type pyocins and the *Photorhabdus luminescens* virulence cassette PVC^20^,^21^,^22^. Variants of this gene cassette equip numerous prokaryotes including pathogenic Gram-negative and Gram-positive bacteria but also Archaea^21^,^23^. Similar to phage tail-like pyocins and type VI secretion systems (T6SS), Afps appear to consist of a contractile sheath, an inner tube, baseplate components and tail fibres, but are devoid of a phage head component (Fig. 1a)^20^,^21^. The genes *Afp1–18* are assumed to encode an ∼100-nm contractile prophage tail structure that on stimulation contracts a sheath protein complex to eject the inner tube, which penetrates the eukaryotic prey cell^20^,^23^,^24^. Afp18 appears to be the toxin unit, the ‘warhead', which is suggested to be enclosed in the core tube of the machinery and injected into eukaryotic host cells^20^. For *Serratia entomophila*, it was shown that Afp18 is responsible for a toxic antifeeding phenotype when introduced into insect hosts^23^.

To study the Afp18 virulence effector of *Y. ruckeri*, we employed the zebrafish embryo as a model, in which cell biological processes during vertebrate development can be visualized at high spatial and temporal resolution. The early cleavage, blastula and gastrula stages of zebrafish development are an excellent model to study individual and collective cellular behaviours^25^. Analyses of infection of zebrafish with *Y. ruckeri* and other pathogens have provided insights into disease mechanism^6^,^26^ as well as function of the innate immune system^27^,^28^.

Here we describe the molecular mechanism of the virulence gene product Afp18 from *Y. ruckeri*. Afp18 mediates toxicity via a glycosyltransferase domain that disrupts RhoA GTPase-dependent actin organization. We find that non-reversible glycosylation at a highly conserved tyrosine residue is responsible for the loss of RhoA activity. Tyrosine glycosylation results in a structural perturbation of the switch I region in RhoA. This inhibits the interaction of RhoA with regulators and downstream signalling effectors, resulting in disturbed actin regulation and abrogates early zebrafish development.

## Results

### Afp18 gene harbours a putative glycosyltransferase domain

**Figure 1:**
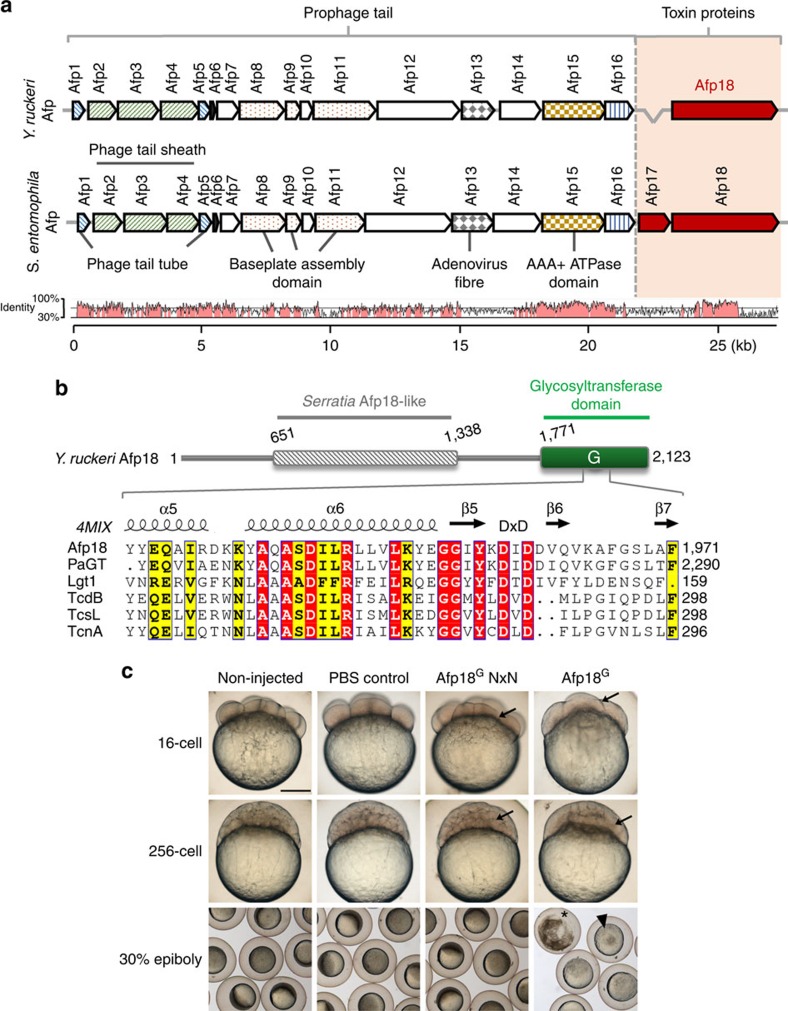
*Yersinia ruckeri* antifeeding prophage tail (Afp) translocation system and effects of the glycosyltransferase domain of Afp18. (**a**) Organization of Afp genes from *Yersinia ruckeri* and *Serratia entomophila* with their predicted and ascribed protein domains. The toxin units are shown in red. Bottom: pairwise genomic gene analysis of aligned Afp gene clusters using mVISTA (http://genome.lbl.gov/vista). Indicated is the average per cent of nucleotide identity within a window of 20 nucleotides. Nucleotide identity >70% is shaded in red. (**b**) Architecture of *Y. ruckeri* Afp18 and similarity to the virulence gene product Afp18 from *S. entomophila*. Amino-acid sequence alignment of the region surrounding the DxD motif (marked) of different toxin glycosyltransferases. Secondary structure elements are deduced from the crystal structure of PaTox (pdb code 4MIX). Accession numbers are the following: *P. asymbiotica* PaTox (PaGT, accession number C7BKP9), *Legionella pneumophila* glucosyltransferase 1 (Lgt1, accession number Q5ZVS2), *Clostridium difficile* toxin B (TcdB, accession number P18177), *C. sordellii* lethal toxin (TcsL, accession number Q46342), *C. novyi* α-toxin (TcnA, accession number Q46149). Alignment was prepared using ClustalW and rendered using ESPript 3.0 (www.espript.ibcp.fr). Identical residues are boxed and shown in red, similar residues are boxed in yellow. (**c**) Live images of non-injected, PBS buffer control, Afp18^G^ NxN and Afp18^G^ protein (each 3 μM, 1 nl injection) injected zebrafish embryos at indicated developmental stages, 16-cell (1.5 h.p.f.), 256-cell (2.5 h.p.f.) and 30% epiboly (4.7 h.p.f.). Arrows mark blastomere cleavage furrows at 16-cell and 256-cell, embryos are oriented animal to the top. At the 16-cell stage, Afp18^G^-injected embryo blastomeres show cleavage planes, while blastomeres fail to establish cell boundaries at the 256-cell stage. Control embryos at 30% epiboly develop normally compared with non-injected WT, while Afp18^G^-injected embryos show disrupted blastoderm (arrowhead) or disintegrate completely (asterisk). Scale bar, 200 μm.

*Y. ruckeri* Afp18 is a component of the prophage tail-like injection machinery (Afp) and exhibits similarities with *Serratia* Afp18 in terms of size and amino-terminal architecture, but differs in the carboxyl-terminal toxic domain (Fig. 1a,b, scheme). The toxic domain of *Yersinia* Afp18 comprises a putative glycosyltransferase domain (Fig. 1b, green coloured region), which exhibits significant sequence similarity to glycosyltransferase toxins from *Legionella pneumophila* (Lgt1–3), *Photorhabdus asymbiotica* (PaTox) and clostridial glycosylating toxins, including toxin A and B from *Clostridium difficile* (Fig. 1b, sequence alignment)^29^. All these glycosyltransferases contain a conserved DxD-(aspartic acid- × -aspartic acid) motif, which is essential for sugar donor substrate binding and, thus, crucial for enzymatic activity. Mutations of this motif result in catalytic defective enzymes^7^,^30^,^31^.

### Afp18^G^ severely affects early zebrafish embryo development

To assess the cellular effects and toxicity of Afp18, we isolated the DNA and cloned the variable carboxyl-terminal fragment (Afp18^G^; amino acids 1,771–2,123) comprising the putative glycosyltransferase domain. In our studies, we used *Y. ruckeri* isolated from an infected rainbow trout in Idaho, USA. This strain is identical to *Y. ruckeri* recently isolated from a wound infection of a 16-year-old male patient in Belgium^32^. We purified the Afp18^G^ protein in *E. coli* and microinjected the recombinant protein into zebrafish zygotes at one-cell stage. In addition, we constructed an Afp18^G^ mutant with an exchange of the DxD motif against an enzymatically non-functional NxN, which we injected as control. Afp18^G^ NxN injected embryos developed normally and were indistinguishable from non-injected or buffer injected control embryos (Fig. 1c). Afp18^G^-injected embryos performed the first three to four cell divisions with morphologically visible cleavage planes between the dividing blastomeres (Fig. 1c, 16-cell stage, arrows), albeit progress of development was delayed compared with controls. At the 256-cell stage, 2.5 h post fertilization (h.p.f.), control embryos showed normal development of the blastoderm positioned on top of the large vegetal yolk cell. In contrast, Afp18^G^-injected embryos failed to establish the typical multilayered organization of the blastoderm and large sections of the blastoderm were devoid of morphologically discernible cell boundaries (Fig. 1c, 256-cell stage, arrow). About 1 h later, at the onset of gastrulation, control embryos initiated epiboly, a coordinated cell movement, in which the static blastomeres became motile and spread vegetalward to cover the yolk cell. In contrast, Afp18^G^-injected embryo did not initiate epiboly, the blastoderm disrupted and in most severe cases the yolk cell and (or) blastomeres lysed and embryos completely disintegrated (Fig. 1c, 30% epiboly, arrowhead and asterisk, respectively). These data reveal that the glycosyltransferase domain of Afp18 is crucial for its severe toxic effect on early zebrafish development.

### Dose-dependent effects of Afp18^G^ on embryo development

**Figure 2:**
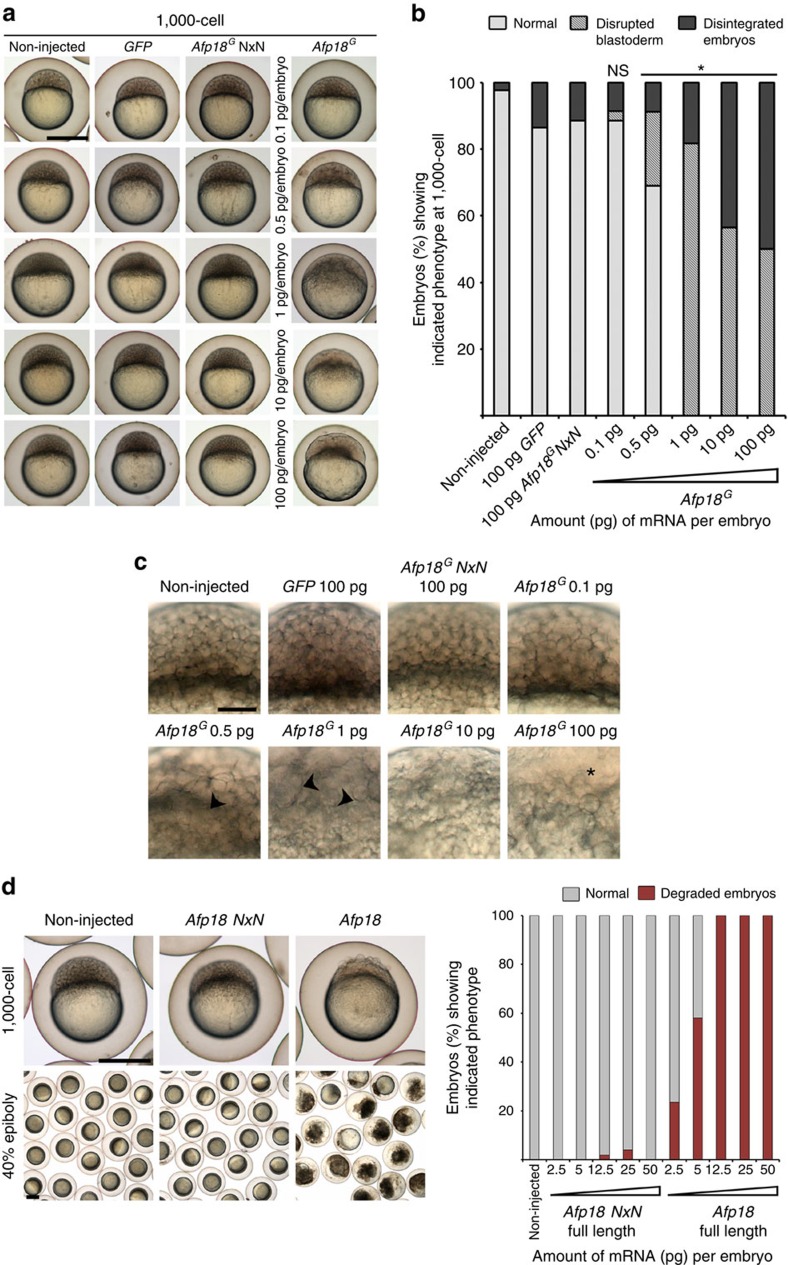
Afp18^G^ disturbs zebrafish early development. (**a**) Live images of non-injected, *GFP*, *Afp18*^*G*^ *NxN* mRNA (each 100 pg per embryo), or different amounts (0.1–100 pg per embryo) of *Afp18*^*G*^ mRNA-injected embryos at 1,000-cell stage (3 h.p.f.). Embryos oriented animal to the top. Scale bar, 500 μm. (**b**) Quantification of the blastoderm phenotype of control and *Afp18*^*G*^-injected embryos shown in **a** at 1,000-cell (non-injected, *n*=41; *GFP* mRNA, *n*=37; *Afp18*^*G*^ *NxN* mRNA, *n*=52; 0.1 pg *Afp18*^*G*^, *n*=35; 0.5 pg *Afp18*^*G*^, *n*=45; 1 pg *Afp18*^*G*^, *n*=60; 10 pg *Afp18*^*G*^, *n*=62; 100 pg *Afp18*^*G*^, *n*=60). Only values for 100 pg per embryo injected *GFP* and *Afp18*^*G*^ *NxN* mRNA are shown. Developing live embryos were classified into categories ‘normal' (WT like), ‘disrupted blastoderm' (cellular structure of blastoderm abnormal) or ‘disintegrated embryos' (blastoderm and (or) yolk cell lysed). The distribution of phenotypes was analysed for significant differences using Fisher exact probability test, revealing significant differences (*P values <0.0001) between *Afp18*^*G*^ *NxN* control and *Afp18*^*G*^ samples. (**c**) Optical image planes of live blastoderm regions at 1,000-cell (3 h.p.f.) of non-injected, *GFP*, *Afp18*^*G*^ *NxN* or *Afp18*^*G*^ mRNA-injected embryos (pg per embryo) oriented animal to the top. Control embryos show normal development of blastomeres, while the blastoderm progressively loses cellular integrity with increasing amounts of *Afp18*^*G*^ mRNA injected. Arrowheads mark the disrupted regions without visible blastomere boundaries, abnormal sized and irregular shaped cells. Asterisks mark detached blastomeres and disintegrated blastoderm. Scale bar, 100 μm. (**d**) Live images of control, full-length *Afp18* and full-length *Afp18 NxN* mRNA (each 50 pg per embryo) injected zebrafish embryos. Afp18-injected embryos disintegrate at 1,000-cell stage (3 h.p.f., upper row, embryos are oriented animal to the top) and degrade early in gastrulation (lower row 40% epiboly, 5 h.p.f.). Scale bar, 500 μm. For five different concentrations each of *Afp18* (non-injected *n*=80; 2.5 pg *n*=34; 5 pg *n*=50; 12.5 pg *n*=65; 25 pg *n*=85; 50 pg *n*=38) and *Afp18 NxN* (2.5 pg *n*=43; 5 pg *n*=58; 12.5 pg *n*=50; 25 pg *n*=76; 50 pg *n*=23) mRNA injections, the bar graph shows percentage of embryos, which at 5 h.p.f. develop like WT controls or degrade.

To rule out contribution of effects by contaminants from the *E. coli*-derived protein preparation, we microinjected mRNA encoding the Afp18^G^ protein. Dilution series of Afp18^G^ encoding mRNA facilitated to determine effects of a wider range of toxin dosage. We injected 0.1–100 pg *in vitro* transcribed *Afp18*^*G*^ or *Afp18*^*G*^ *NxN* mRNA per embryo, or as control *GFP* mRNA. Morphological phenotypes were documented at the 1,000-cell stage (3.3 h.p.f.; Fig. 2a,b quantitative analysis; Supplementary Fig. 1). Embryos injected with 0.1 pg *Afp18*^*G*^ mRNA did not develop significantly different from control embryos. About 30% of the embryos injected with 0.5 pg *Afp18*^*G*^ mRNA developed a disintegrated blastoderm, with irregular cell shape, local loss of blastomere boundaries and abnormal cell sizes (arrowhead in Fig. 2c, 0.5 pg). Injection of 1 pg *Afp18*^*G*^ mRNA per embryo affected cell morphology in all analysed embryos. Frequently, larger cells detached from the blastoderm and abnormal vesicles were formed (arrowhead in Fig. 2c). When 10 or 100 pg *Afp18*^*G*^ mRNA were injected per embryo, cell morphology was severely affected, tissue integrity progressively lost during gastrulation and blastoderms frequently disintegrated (Fig. 2c and Supplementary Fig. 1a). In contrast, microinjection of mRNA encoding the glycosyltransferase-deficient mutant Afp18^G^ NxN had no effect on embryonic development, revealing that the effects were indeed caused by the glycosyltransferase activity. Given that the effects of Afp18^G^ on cellular functions cannot be analysed when embryos disintegrate early, we chose to inject 0.5 pg *Afp18*^*G*^ mRNA for further analyses.

### The G domain is the major pathogenicity determinant in Afp18

Next we wanted to clarify whether parts of Afp18 other than the glycosyltransferase (G) domain affect zebrafish embryo development. Therefore, we injected mRNA encoding the full-length Afp18 protein, or the enzymatically non-functional Afp18 NxN mutant, and analysed embryos and early larvae to identify potential morphological alterations in embryogenesis. Full-length Afp18 caused severely degraded embryos during early gastrulation (40% epiboly; Fig. 2d), while *Afp18 NxN* injected embryos developed indistinguishably from non-injected wild-type (WT) control embryos (Fig. 2d). These data revealed that the glycosyltransferase domain is the Afp18 protein domain mediating toxicity in this assay.

### Afp18^G^ disrupts the actin cytoskeleton

**Figure 3:**
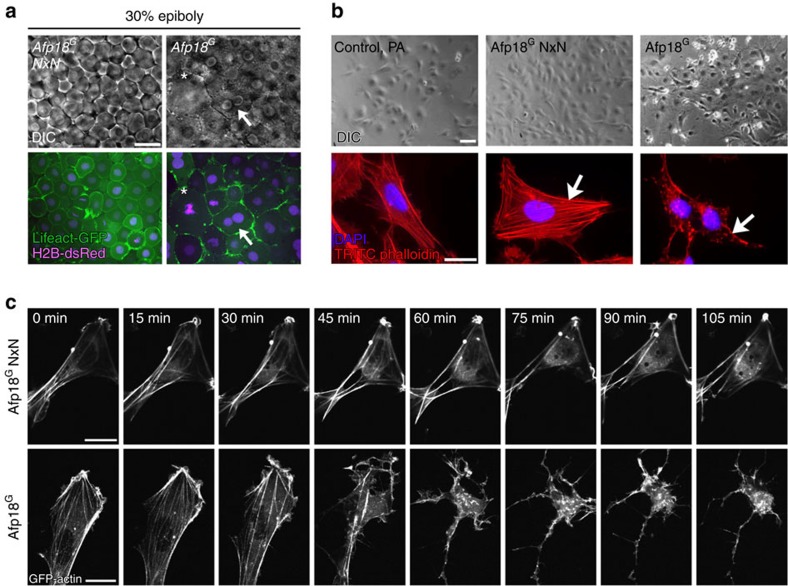
Afp18^G^ affects the actin cytoskeleton. (**a**) Live images of *Afp18*^*G*^ *NxN* or *Afp18*^*G*^ mRNA (each 0.5 pg per embryo) injected embryos at 30% epiboly. Embryos are co-injected with mRNA encoding *Lifeact-GFP* (green) and *H2B-dsRed* (magenta; 100 pg per embryo each) labelling the F-actin cytoskeleton and the nuclei. *Afp18*^*G*^ *NxN* embryos developed indistinguishable from WT embryos. Differential interference contrast (DIC) images of blastomeres are shown in the upper row, the corresponding confocal epifluorescence images below. Asterisk marks abnormally large blastomere; the arrow marks a blastomere with two nuclei. Single-plane image, scale bar, 20 μm. (**b**) Fluorescent micrographs of ZF4 cells treated with 6xHis-tagged Afp18^G^ (right panel) or glycosyltransferase-deficient mutant Afp18^G^ NxN (middle panel) proteins in combination with anthrax protective antigen (PA) as translocation system for His-tagged proteins. Top row shows phase-contrast images. Bottom row shows TRITC-phalloidin staining (red) of the actin cytoskeleton and a DAPI nuclei staining (magenta) of ZF4 cells fixed after 2 h. Arrows indicate regular stress fibres of F-actin in Afp18^G^ NxN cells compared with disrupted F-actin fibres in Afp18^G^ treated cells. Scale bar, 50 μm (top panel), 10 μm (bottom panel). (**c**) Time-lapse microscopic images of GFP-actin expressing HeLa cells intoxicated with Afp18^G^ NxN (top row) and Afp18^G^ (bottom row) as described in **b**. Scale bar, 10 μm. DAPI, 4′,6-diamidino-2-phenylindole.

To analyse the *in vivo* cellular components affected by the Afp18^G^ toxin, we co-injected mRNAs encoding Lifeact-GFP (green fluorescent protein) to fluorescently label the actin cytoskeleton and histone H2B-dsRed to label nuclei in living embryos. On *Afp18*^*G*^ mRNA injection, blastomeres were severely enlarged (Fig. 3a, asterisk). In addition, blastomeres frequently contained two or more nuclei. We assumed that these Afp18^G^-induced morphological alterations were caused by deregulation of F-actin cytoskeleton organization. The Lifeact-GFP signal suggested a reduced amount of polymerized actin in *Afp18*^*G*^-expressing embryonic cells (Fig. 3a, Supplementary Movie 1). To analyse the effects of Afp18^G^ on the actin cytoskeleton in more detail, we introduced recombinant 6xHis-tagged glycosyltransferase domain protein into zebrafish ZF4 cells (embryo-derived fibroblast-like cell line)^33^, using protective antigen (PA; the binding and translocation component of anthrax toxin) as a delivery system^34^, and stained filamentous actin with TRITC-phalloidin. Cells to which the glycosyltransferase-deficient NxN-mutant was delivered (Fig. 3b, middle panel) had an actin cytoskeleton indistinguishable from untreated control cells. In contrast, cells to which Afp18^G^ was delivered showed a massive loss of actin fibres (Fig. 3b, bottom right panel) and finally rounded up (Fig. 3b top right panel). Live imaging of ZF4 cells transfected with GFP-actin revealed a complete disassembly of the actin cytoskeleton after delivery of Afp18^G^ (Fig. 3c, time series bottom row; top row Afp18^G^ NxN-treated control cell). The actin-depolymerizing effect was remarkably rapid. During 60 min of incubation, actin fibres disappeared and ZF4 cells collapsed. Comparable effects were observed using human HeLa cells (Supplementary Fig. 3a). Thus, the glycosyltransferase activity of Afp18 appears to severely affect the regulation of cellular actin.

### Afp18^G^ affects cytokinesis in early development

**Figure 4:**
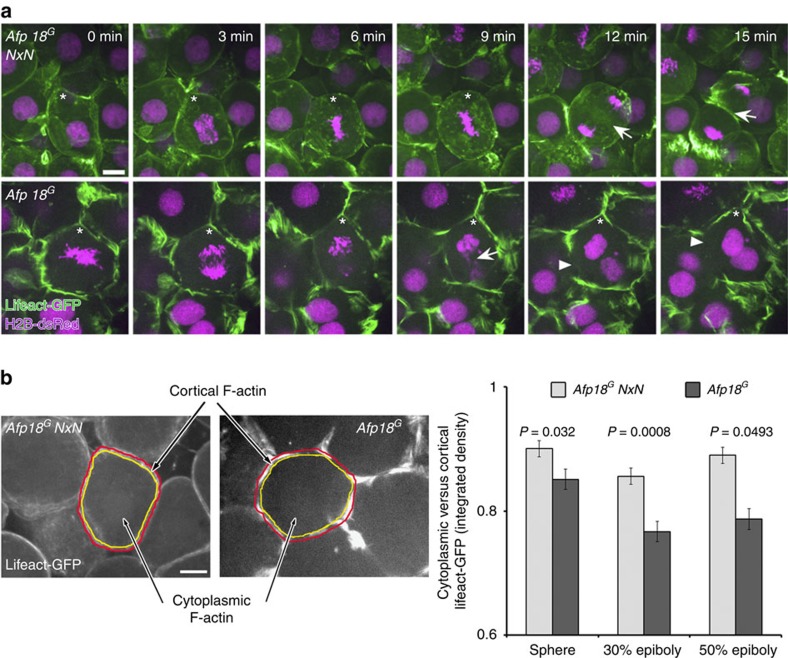
Afp18^G^ blocks cytokinesis leading to multinucleated cells. (**a**) Cytoskeleton and cytokinesis analysed by time series of *Afp18*^*G*^ *NxN* (upper row) or *Afp18*^*G*^ (lower row) mRNA (0.5 pg per embryo each) injected embryos at dome stage (4.3 h.p.f.). Embryos were co-injected with mRNA encoding *Lifeact-GFP* (green) and *H2B-dsRed* (magenta; 100 pg per embryo each) labelling the F-actin cytoskeleton and the nuclei. Asterisk marks dividing blastomeres. *Afp18*^*G*^ *NxN* and *Afp18*^*G*^ mRNA-injected blastomeres complete microtubule mediated mitotic phases including chromosome segregation. Arrows indicate the actin ring contraction during cytokinesis, which is strongly affected in *Afp18*^*G*^-dividing blastomeres. Confocal *z*-stack projection of 10-μm depth; scale bar, 10 μm. (**b**) Quantification of cytoplasmic versus cortical Lifeact-GFP integrated epifluorescence signal density at indicated developmental stages. Confocal image of *Afp18*^*G*^ *NxN* (left side) and *Afp18*^*G*^ (right side) injected embryo showing an example blastomere with manually defined areas of cortical (region depicted in between red and yellow selection) and cytoplasmic (region outlined by yellow selection) F-actin and analysed using Fiji-ImageJ software measure function (sphere, *n*=12 each; 30% epiboly *n*=12 each; 50% epiboly *n*=12 each). Scale bar, 10 μm. Values are average±s.e.m. Statistical significance was evaluated using Mann–Whitney test.

We examined whether the completion step of cell division, involving coordinated actin rearrangement, was affected by Afp18. We found that cytokinesis proceeded normally in *Afp18*^*G*^ *NxN* expressing control embryos (Fig. 4a, upper row, arrow; Supplementary Movie 1). In contrast, cytokinesis including the assembly of the actomyosin ring and formation of the cleavage furrow was severely impaired in blastomeres of *Afp18*^*G*^ mRNA-injected embryos, resulting in cells frequently containing more than one nucleus (Fig. 4a, lower row, arrow; Supplementary Movie 1). We evaluated cortical and cytoplasmic deposition of F-actin via measurement of the integrated density of Lifeact-GFP fusion protein fluorescence (Fig. 4b). The ratio of cytoplasmic versus cortical F-actin localization at three developmental time points (sphere, 30% epiboly, 50% epiboly) was almost constant in blastomeres of *Afp18*^*G*^ *NxN* control injected embryos, when measured in 30 min time windows. In contrast, cortical actin was significantly enriched in blastomeres of *Afp18*^*G*^ mRNA-injected embryos, indicating a deregulation of the dynamic organization of the actin cytoskeleton. However, the cell cycle phases involving proper assembly and organization of microtubule-based mitotic spindle progressed normally. Time series of Lifeact-GFP and H2B-dsRed labelled embryos revealed the accurate composition of the metaphase, with the sister chromatids moved to the opposite spindle poles for both, *Afp18*^*G*^ *NxN* (Fig. 4a, upper row) and *Afp18*^*G*^ (Fig. 4a lower row) mRNA-injected embryos. In summary, Afp18^G^ seemed to selectively affect actin-dependent filament organization and dynamics during cytokinesis, while microtubule dynamics and karyokinesis appeared to progress normally.

### Bleb formation is impaired by Afp18^G^

**Figure 5:**
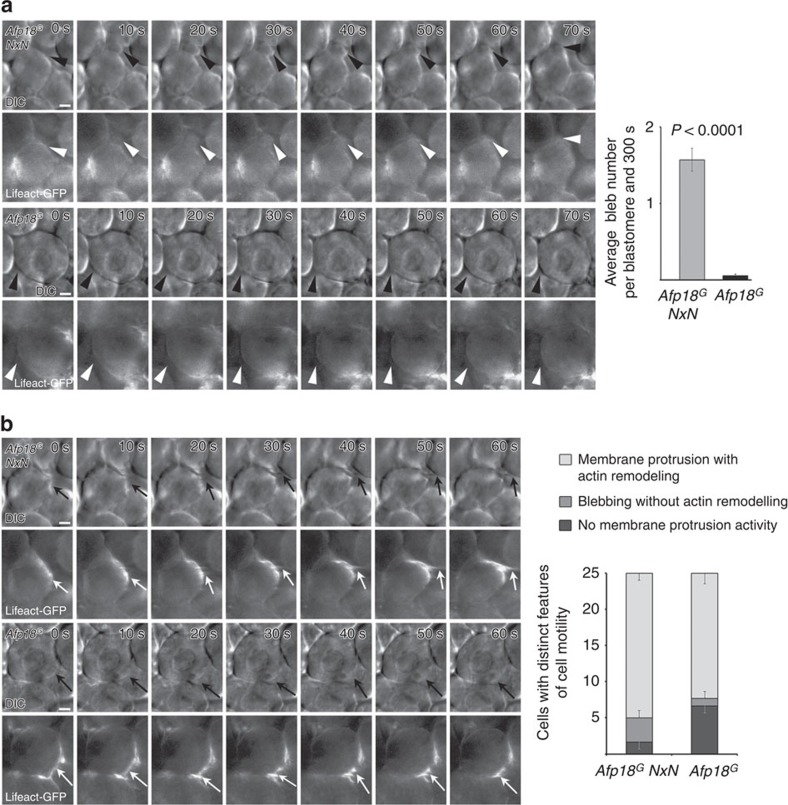
Afp18^G^ blocks cell blebbing without affecting lamellipodia and filopodia formation. (**a**) Bleb formation analysed at 30% epiboly (4.7 h.p.f.) by time series of embryos injected with 0.5 pg per embryo of *Afp18*^*G*^ *NxN* mRNA (upper two rows) or *Afp18*^*G*^ mRNA (lower two rows). Embryos were co-injected with mRNA encoding *Lifeact-GFP* (100 pg per embryo) labelling the F-actin cytoskeleton. DIC images are shown in the upper row and corresponding epifluorescence images in the lower row. Black arrowheads indicate spherical membrane protrusions of a blastomere, known as bleb formation, which are initially devoid of actin (white arrowheads). In contrast, blastomeres of *Afp18*^*G*^-injected embryos show significantly less blebbing and blebs appear smaller. Graph shows the quantification of bleb formation of blastomeres in *Afp18*^*G*^ mRNA compared with *Afp18*^*G*^ *NxN* control injected embryos (0.5 pg per embryo) during a time series (frames: 10-s intervals for 300 s; *Afp18*^*G*^ *NxN n*=75; *Afp18*^*G*^ *n*=75 cells analysed of three different embryos, 25 blastomeres each). Values are average±s.e.m.; statistical significance was analysed using Mann–Whitney test. Scale bar, 10 μm. (**b**) Protrusive activity analysed in time series of *Afp18*^*G*^ *NxN* (upper two rows) or *Afp18*^*G*^ (lower two rows) mRNA (0.5 pg per embryo each) injected embryos at 30% (4.7 h.p.f.). Embryos were co-injected with mRNA encoding *Lifeact-GFP* (100 pg per embryo) labelling the F-actin cytoskeleton. DIC images are shown in rows 1 and 3 and corresponding fluorescent image in the rows 2 and 4. Both *Afp18*^*G*^ *NxN* and *Afp18*^*G*^ mRNA-injected embryos show blastomeres forming sheet-like membrane protrusions resembling lamellipodia, filled with actin bundles and actin branches (arrows). Scale bar, 10 μm. Quantification shows the percentage of analysed blastomeres forming blebs without actin, blastomeres forming sheet-like membrane protrusions with actin or blastomeres without membrane protrusion activity during the captured time series (*Afp18*^*G*^ *NxN n*=41; *Afp18*^*G*^ *n*=37 cells analysed from three different embryos). The statistical significance (*P*=0.0091) of differences between the proportion of *Afp18*^*G*^ *NxN* and *Afp18*^*G*^ blastomeres showing these specific behaviours was computed using the Kruskal–Wallis test. The analysis revealed that the distribution of cell behaviour types indeed differs significantly, caused by changes in blebbing activity but not in lamellipodia formation. DIC, differential interference contrast.

The organization of the cortical actin network controls cell membrane protrusions, and, thus, has a strong influence on cell motility^35^,^36^. It was shown that spherical protrusions, called blebs, are formed dynamically at the membrane of migrating cells. We scored bleb formation of blastomeres of *Afp18*^*G*^ and *Afp18*^*G*^ NxN mRNA-injected embryos at the onset of epiboly. Fig. 5a and Supplementary Movie 2 show a time series of a forming bleb documented by differential interference contrast transmitted light (upper row, black arrowhead) and fluorescence microscopy of Lifeact-GFP (lower row, white arrowhead) of a control injected embryo (upper two rows). The three phases of a bleb life cycle—initiation (after 10 s), expansion (up to 30 s) and retraction (40 to 70 s)—were clearly visible. Initially, blebs form and grow devoid of actin, while during retraction fluorescently labelled Lifeact-GFP-actin signal appeared slightly enhanced. Blebbing was severely reduced in numbers but also in bleb size in *Afp18*^*G*^ mRNA-injected embryos (Fig. 5a lower two rows), as quantified by bleb counts (Fig. 5a graph). In addition, we analysed other actin-driven protrusion behaviours of blastomeres, excluding blebbing. Time series of both *Afp18*^*G*^ and *Afp18*^*G*^ *NxN* mRNA-injected embryos showed blastomeres, which form lamellipodia driven by actin remodelling (marked by black and white arrows in differential interference contrast and fluorescence images, respectively; Fig. 5b and Supplementary Movie 2). Quantification revealed that blastomeres did not significantly differ in lamellipodia number (membrane protrusions with actin remodelling—see graph in Fig. 5b) when *Afp18*^*G*^ and *Afp18*^*G*^ *NxN* expressing embryos were compared.

Blebs have been reported to be primarily controlled by RhoA and its effector ROCK-I^37^,^38^. In contrast, Rac and Cdc42, which increase actin-dependent lamellipodia formation, inhibit bleb formation and amoeboid migration^39^,^40^. Therefore, our *in vivo* data suggested that Afp18^G^ predominantly targeted RhoA, whereas Rac1 and Cdc42 might be less affected. To determine whether Afp18 may co-localize with RhoA, we used DNA vector injection to generate mosaic embryos in which individual cells were expressing enzyme-deficient EGFP-tagged Afp18^G^ NxN and RHOA. Anti-EGFP and anti-RHOA immunofluorescence revealed that EGFP-Afp18^G^ NxN appeared to target the cell membrane, and evaluation of line scans of fluorescent profiles shows co-localization with RHOA (Supplementary Fig. 2).

### Afp18^G^ utilize UDP-*N*-acetylglucosamine to modify Rho GTPases

**Figure 6:**
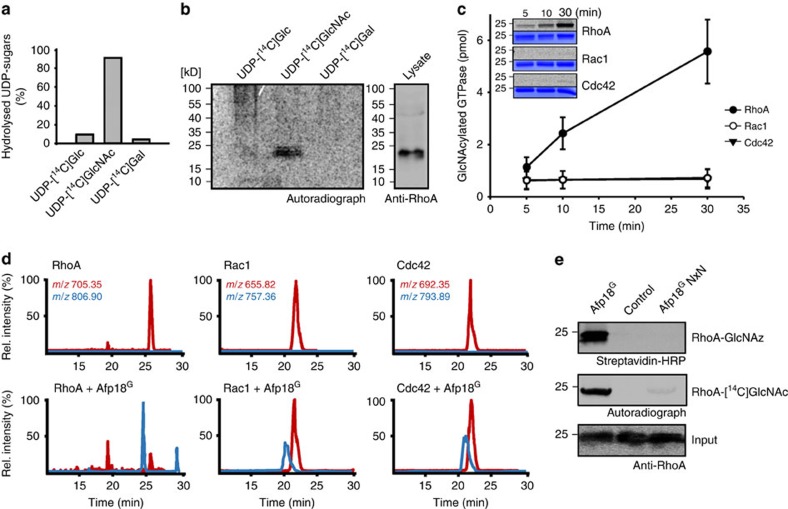
Afp18^G^ selectively modifies RhoA using UDP-GlcNAc. (**a**) Donor substrate specificity of Afp18^G^ determined by UDP-glycosidase activity. Percentage of hydrolysed UDP-[^14^C]sugars was determined by PEI thin-layer chromatography and autoradiography after incubation for 10 min at 30 °C. Data are representative of three independent experiments. (**b**) Autoradiography of the SDS–PAGE from cell lysate incubated with Afp18^G^ and indicated radiolabeled UDP-sugars. Western blot of RhoA (right panel) showed similar electrophoretic mobility as radiolabelled proteins. (**c**) Time course of *in vitro* GlcNAcylation of RhoA, Rac1 and Cdc42 by Afp18^G^ (1 nM). Inserts show representative autoradiograms (upper panel) and Coomassie-stained SDS–PAGE (bottom panel). Error bars indicate s.e.m.'s of three technical replicates. (**d**) Extracted ion chromatograms of thermolysin-digested GST-RhoA, GST-Rac1 and Cdc42 treated with Afp18^G^ (lower chromatogram) or untreated control (upper chromatogram). The molecular mass [M+2H]^2+^ of the switch I peptides (RhoA: _26_SKDQFPEV**Y**VPT_37_; Rac1: _24_TTNAFPGE**Y**IPT_35_; Cdc42: _24_TTNKFPSE**Y**VPT_35_) are indicated. Afp18^G^-modified GTPases (lower chromatogram) show a mass shift of 203 Da revealing a modification with a single *N*-acetylglucosamine (shifted blue curves). In comparison with Rac1 and Cdc42, RhoA was modified most efficiently. (**e**) The DxD motif of Afp18 is crucial for GlcNAcylation of RhoA. Recombinant RhoA was incubated in the presence UDP-GlcNAz or UDP-[^14^C]GlcNAc with Afp18^G^, Afp18^G^ NxN or without toxin. Modified proteins were analysed by click chemistry using biotin alkyne and western blotting and autoradiography, respectively. Anti-RhoA served as input control.

To identify the cellular targets of Afp18 in zebrafish, we elucidated the sugar donor for this reaction by enzyme-catalysed UDP-[^14^C]sugar hydrolysis and found that Afp18^G^ efficiently hydrolysed UDP-[^14^C]GlcNAc (Fig. 6a). Using UDP-[^14^C]GlcNAc in a glycosylation reaction with Afp18^G^ and cell lysate, we identified proteins with an electrophoretic mobility corresponding to 23 kDa, which were labelled with ^14^C-GlcNAc and migrated similarly to the GTPase RhoA (Fig. 6b). As Rho GTPases are known regulators of the actin cytoskeleton and putative substrate candidates, we applied RhoA, Rac1 and Cdc42 to an *in vitro* glycosylation reaction with 1 nM Afp18^G^ and obtained a strong modification of RhoA. Signals of Rac1 and Cdc42 were hardly visible under these conditions (Fig. 6c). When we applied higher amounts of Afp18^G^ (100 nM), we could also observe the glycosylation of RhoB, RhoC, Rac2 and Rac3, and Cdc42 (Supplementary Fig. 3b). Other subfamily members of the Rho family were not modified and also Ras proteins did not serve as substrates. Using mass spectrometric analysis of Afp18^G^ glycosylated RhoA, Rac1 and Cdc42, we could confirm that these GTPases were modified by a single covalently attached *N*-acetylhexosamine (HexNAc) moiety, which resulted in a mass increase of 203 Da in the corresponding switch I peptides (Fig. 6d). Again, RhoA was modified most efficiently among these GTPases. Thus, RhoA may be the primary target of Afp18^G^ and might explain the previous results obtained in RhoA-, Rac1- and Cdc42-mediated actin dynamic analysis in zebrafish embryos and ZF4 cells. To prove that the NxN mutant of Afp18 is inactive and not able to glycosylate RhoA, we used UDP-GlcNAz as donor in a click chemistry reaction with biotin alkyne and radiolabelled UDP-[^14^C]GlcNAc in a glycosylation reaction and show that the mutant indeed is deficient in glycosyltransferase activity (Fig. 6e). Furthermore, we analysed the nucleotide status of RhoA which is glycosylated by Afp18 and found that Afp18^G^ preferentially modified GTP (GTPγS)-bound RhoA in comparison to GDP-bound or nucleotide-free RhoA (Supplementary Fig. 4a).

### GlcNAcylated RhoA is observed in an inhibited conformation

**Figure 7:**
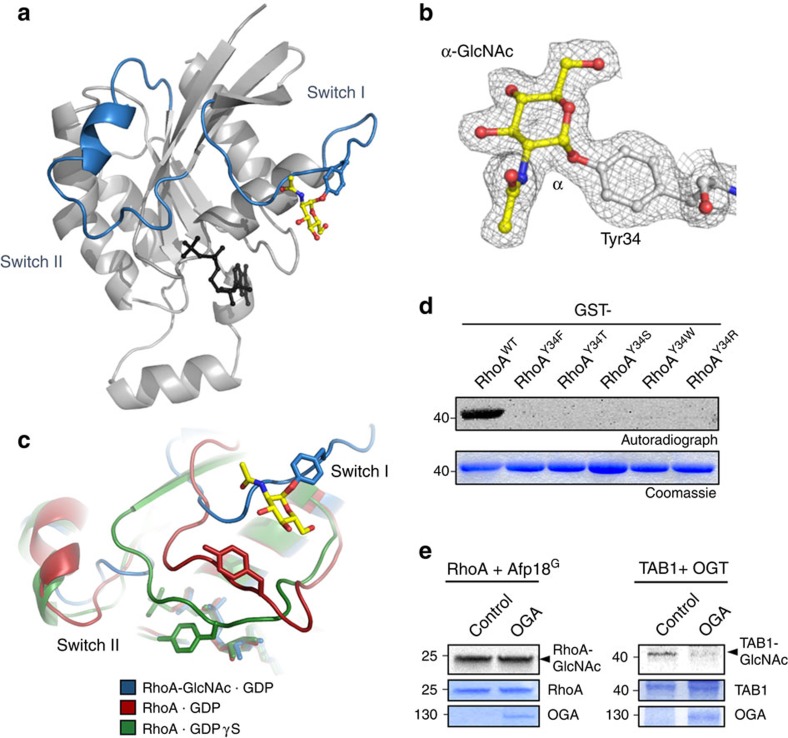
Structural consequences of RhoA GlcNAcylation at tyrosine-34. (**a**) Crystal structure of glycosylated RhoA at tyrosine-34. Switch I and switch II regions are highlighted in blue. GDP is shown as sticks and balls in black. The GlcNAc moiety attached to tyrosine-34 is shown as sticks and balls in yellow. (**b**) Electron density map of a section of 2F_o_−F_c_ protein (grey), contoured at a level of 1*σ*, showing GlcNAc moiety attached to tyrosine-34 of RhoA in the alpha configuration of the glycosidic bond (marked). Additional residues of the switch I region were omitted for clarity. (**c**) Superposition of the effector loops of GlcNAcylated RhoA with structures of non-glycosylated active GTPγS-bound RhoA (pdb code 1A2B)^42^ and inactive GDP-bound RhoA (pdb code 1FTN)^41^. The switch I and II regions of GlcNAc-modified RhoA adopt distinct open conformations. (**d**) Autoradiograms and Coomassie stainings of Afp18^G^-catalysed *in vitro*^14^C-GlcNAcylation of WT GST-RhoA and the indicated mutants. (**e**) Cytoplasmic OGA is unable to revert GlcNAcylation of Rho. RhoA was radioactively preglycosylated by Afp18^G^ and applied to a deglycosylation reaction with GST-OGA. TAB1, preglycosylated by OGT, served as positive control. Autoradiographs (top panels) of ^14^C-GlcNAcylated RhoA and GlcNAcylated TAB1 are shown. Coomassie staining of RhoA, TAB1 and GST-OGA are shown as input controls.

To identify the site of modification and gain insights into the structural alteration caused by GlcNAcylation of RhoA, we crystallized Afp18-modified RhoA in the presence of magnesium and GDP and solved the X-ray structure at 2.0 Å resolution (Fig. 7a, Table 1). In the RhoA structure, we could clearly assign additional electron density at the hydroxyl group of tyrosine-34 to an attached *N*-acetylglucosamine moiety (Fig. 7b, Supplementary Fig. 3c). Interestingly, despite the presence of magnesium in the crystallization conditions, the structure revealed an unusual GDP-bound but magnesium-free conformation. The overall fold of the crystal structure of GlcNAcylated RhoA is very similar to known structures of Rho GTPases. However, the switch regions, especially the switch I region, adopt a conformation with tyrosine-34 positioned away from the nucleotide binding pocket, resulting in a structure distinct from the structures of RhoA bound to GDP^41^ or GTP^42^, which is generally not compatible with effector and regulator interaction (Fig. 7c).

Tyrosine-34 is located within the effector loop region conserved in all Rho family GTPases. In *in vitro* glycosylation experiments with Afp18, followed by tandem mass spectrometric (LC–MS–MS) analyses, we confirmed that also Cdc42 was GlcNAcylated at tyrosine-32 (Supplementary Fig. 3d). Furthermore, site-directed mutagenesis and *in vitro* glycosylation experiments with Y32(34)F mutants of RhoA, Rac1 and Cdc42 confirmed switch I tyrosine-32(34) as the acceptor site of modification (Fig. 7d, Supplementary Fig. 3e). No other hydroxyl-containing amino acid (threonine or serine) or recently discovered glycosyl acceptor residues as tryptophan^43^ or arginine^44^,^45^ were able to substitute tyrosine in RhoA as an acceptor residue for glycosylation (Fig. 7d).

### Afp18 forms deglycosylation-resistant α-glycosidic bonds

The defined electron density of the sugar attached to the hydroxyl group of tyrosine-34 revealed the α-anomeric configuration of the glycosidic bond (Fig. 7b). This finding implies that the glycosylation mechanism proceeds under retention of the stereochemistry of D-α-GlcNAc. Thus, Afp18 can be grouped into the family of retaining glycosyltransferases. The stereochemistry of the glycosidic bond has most likely no influence on the functional consequences of Afp18-mediated glycosylation, but might have an influence on the stability of the glycoside inside the host cytoplasm. Only one enzyme, namely *O*-GlcNAcase (OGA) exists in eukaryotic cytoplasm, which is able to remove mono-*O*-GlcNAc moieties from proteins. OGA predominantly cleaves sugars attached in the β-configuration. To clarify whether the glycosidic bond on RhoA was resistant to hydrolysis of OGA, we tested ^14^C-GlcNAcylated RhoA in a deglycosylation reaction. As a control protein, we used TGF-beta-activated kinase 1 (TAB1) preglycosylated by nucleocytoplasmatic *O*-GlcNAc transferase (OGT), which was efficiently deglycosylated by OGA (Fig. 7e). Notably, OGA did not deglycosylate Afp18-modified RhoA. We confirmed these results by using CpNagJ, another highly efficient mono-*O*-GlcNAcase from *Clostridium perfringens* (Supplementary Fig. 3f,g). Taken together, we found that Afp18 is a mono-*O*-glycosyltransferase, which selectively uses UDP-GlcNAc as sugar donor substrate to modify Rho GTPases site-specifically at a switch I tyrosine residue.

### RhoA GlcNAcylation blocks regulator and effector interaction

**Figure 8:**
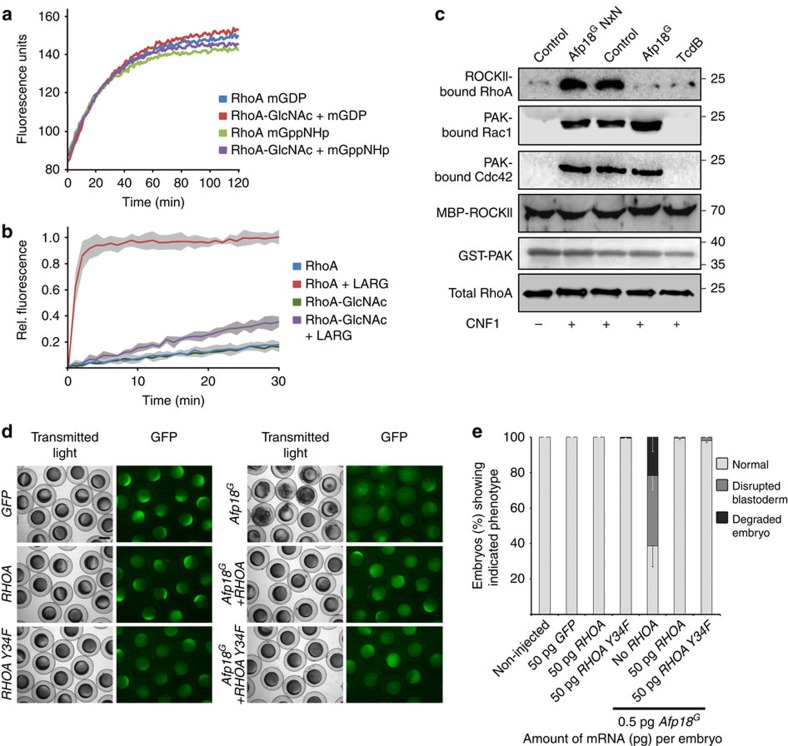
Functional consequences of tyrosine-34 glyosylation of RhoA. (**a**) GlcNAcylation of RhoA by Afp18^G^ does not alter nucleotide binding. Fluorimetric analysis of mant-GDP (mGDP) or mant-GppNHp (mGppNHp) binding to WT RhoA or glycosylated RhoA (RhoA-GlcNAc) bound to GDP. Nucleotide exchange was monitored by the increase in fluorescence on mant-nucleotide binding to RhoA. Data are representative of two independent experiments. (**b**) Nucleotide exchange was measured by mant-GppNHp exchange with WT RhoA, Afp18^G^-GlcNAcylated RhoA in the presence or absence of LARG. Data are represented as means±s.d. of three technical replicates. (**c**) Western blot analysis of RhoA, Rac1, Cdc42 pull-down experiments with zebrafish (ZF4) cells treated with Afp18^G^ (plus PA for delivery), Afp18^G^ NxN (plus PA for delivery) and *C. difficile* toxin B (TcdB). After Rho GTPase activation with CNF1, active RhoA was pulled down with ROCK II-coupled beads and Rac1 and Cdc42 with PAK-coupled beads. Bound GTPases were detected by western blotting using anti-RhoA, anti-Rac1, and anti-Cdc42 antibodies, respectively. Immunoblot of total RhoA is the loading control. (**d**) Live images at 30% epiboly (4.7 h.p.f.) of control non-injected, *GFP* mRNA (50 pg per embryo), *RHOA* mRNA (50 pg per embryo), *RHOA Y34F* (50 pg per embryo)+*Afp18*^*G*^ mRNA (0.5 pg per embryo), *Afp18*^*G*^ mRNA (0.5 pg per embryo)+*RHOA* mRNA (50 pg per embryo) or *Afp18*^*G*^ (0.5 pg per embryo)+*RHOA Y34F* mRNA (50 pg per embryo) injected embryos. *GFP* mRNA (50 pg per embryo) was also included in each injection mix, and GFP fluorescence used to examine for homogenous expression: panels show transmitted light images in left columns and corresponding GFP fluorescence images in right columns. *GFP*, *RHOA* and *RHOA Y34F* injected embryos developed indistinguishable from non-injected control embryos. Scale bar, 500 μm. (**e**) Quantification of percentage embryos that develop normally or were severely affected regarding the organization of the blastoderm in experiment shown in **d**. Co-injection of *RHOA* or *RHOA Y34F* mRNA together with *Afp18*^*G*^ mRNA significantly reduces the fraction of disrupted and degraded blastoderm phenotypes, and increases the fraction of embryos developing normally at 30% epiboly. Values are average mean±s.e.m. of four biological replicates. Significance (*P* values <0.001) of changes in phenotype distribution was valuated using the Kruskal–Wallis test.

To elucidate the molecular consequences of tyrosine-34 GlcNAcylation of RhoA, we analysed the nucleotide binding and nucleotide exchange of RhoA by mant-GDP fluorescence spectroscopy. Whereas the Afp18-catalysed glycosylation of RhoA did not affect nucleotide binding kinetics of mant-GDP or mant-GppNHp, a non-hydrolysable GTP analogue, (Fig. 8a), RhoA modification blocked leukaemia-associated RhoGEF (LARG)-catalysed nucleotide exchange (Fig. 8b). This effect was not caused by competition of Afp18^G^ with GEF (Supplementary Fig. 4b), because GEF-stimulated nucleotide exchange was only blocked by the addition of UDP-GlcNAc. Thus, RhoA GlcNAcylation by Afp18 prevented the activation step of the GTPase. In addition, the interaction of RhoA to its GTPase-activating protein p50RhoGAP was impaired by Afp18^G^-mediated glycosylation of tyrosine-34 (Supplementary Fig. 4c).

Next, we analysed the interaction of Rho, Rac1 and Cdc42 with its downstream effectors Rho kinase α (ROCKII)- and p21-associated kinase (PAK) and used zebrafish (ZF4) cells in an effector pull-down assay using ROCKII- and PAK-coupled beads (Fig. 8c). To activate Rho GTPases independently of intracellular GEFs, we pretreated cells with cytotoxic necrotizing factor 1 (CNF1), a toxin that activates Rho GTPases constitutively by deamidation^46^. Subsequent cell intoxication with Afp18^G^ totally blocked RhoA interaction with ROCKII, whereas RhoA could efficiently be precipitated from cells treated with the glycosyltransferase-deficient mutant Afp18^G^ NxN. Toxin B from *Clostridium difficile* (TcdB), which glycosylates threonine-37 in RhoA and thereby inhibits Rho effector interaction, served as a control. Impaired effector interaction catalysed by Afp18^G^ was also observed for Rhotekin, an effector of RhoA, in human HeLa cells (Supplementary Fig. 4d). Intriguingly, the interaction of Rac1 or Cdc42 with their effector PAK could not be blocked by Afp18-mediated GlcNAcylation, whereas TcdB treatment of ZF4 cells efficiently prevented PAK interaction (Fig. 8c). This is consistent with our findings in time-lapse microscopy of Afp18-intoxicated ZF4 cells, which showed a rapid degradation of filamentous stress fibres but a persistence of membrane dynamics like membrane ruffling and filopodia formation, which are regulated by Rac1 and Cdc42, respectively (Fig. 3c, Supplementary Movie 3). It seems that Afp18 specifically inactivates RhoA signalling, but less signalling of Rac or Cdc42.

### Afp18^G^-phenotype suppression by RhoA overexpression

To determine whether Afp18^G^ predominantly acts through the glycosylation of RhoA *in vivo*, we tried to rescue the toxin phenotype by co-injection of mRNA encoding human WT RHOA or the non-glycosylatable mutant version RHOA Y34F. Zebrafish RhoA as well as other Rho GTPases share over 95% sequence conservation with their human homologues^16^. Embryos injected with human *RHOA* or *RHOA Y34F* mRNA (up to 50 pg per embryo) alone developed normal, indistinguishable from non-injected or *GFP* mRNA-injected control embryos (Supplementary Fig. 5a). We co-injected *RHOA* or *RHOA Y34F* mRNA with 0.5 pg *Afp18*^*G*^ mRNA and found the disrupted blastoderm phenotype rescued to a large extent, with more embryos developing normally compared with *Afp18*^*G*^ mRNA-injected embryos (Fig. 8d and quantification Fig. 8e). Taken together, both human *RHOA* and *RHOA Y34F* overexpression were able to rescue development of early embryos from Afp18^G^ toxicity.

## Discussion

Here we unravelled the mode of action of the toxic effector Afp18 of the prophage tail-like protein translocation machinery Afp from *Y. ruckeri*, which is the causative agent of enteric redmouth disease in salmonid fish species. We employed Afp18 for studies in zebrafish embryos, which have been shown to be a highly sensitive fish model for effector and toxin analyses^26^,^27^,^28^,^47^.

We observed that microinjection or expression of *Afp18* in zebrafish early embryos abrogated development, and embryos died before gastrulation was completed. The glycosyltransferase-deficient mutant (*Afp18 NxN*) showed no developmental defects unravelling the glycosyltransferase domain as a major toxic determinant. Even at low expression of the glycosyltransferase domain Afp18^G^ alone (0.1 pg *Afp18*^*G*^ mRNA), cytokinesis was severely affected and multinucleated cells were formed. While microtubule-dependent processes like karyokinesis, including metaphase formation and chromatid segregation, progressed normally, actin filament dependent processes (for example, cytokinesis) were blocked. This finding became even more obvious by cell behaviour defects observed during gastrulation, when cells become motile. *Afp18*^*G*^-expressing blastomeres contained significantly less cytoplasmic filamentous actin compared with controls.

Notably, the *Yersinia* effector-expressing blastomeres had severely reduced blebbing activity. Membrane blebbing depends on actomyosin and is essential for amoeboid cell migration^37^,^38^,^39^,^48^. Accordingly, the combined reduction of blebbing and protrusive activity effectively blocked cell movements and progress of gastrulation, and finally abrogated embryo development. Deduced from the specific effects of Afp18 on the actin cytoskeleton (for example, inhibition of blebbing), we proposed that the observed defects of zebrafish embryo development depend on an action of Afp18^G^ on the GTP-binding protein RhoA. This hypothesis was supported by numerous previous reports, showing that RhoA and its effector ROCK are essential for bleb formation and for amoeboid migration^37^,^38^. Notably, Rac and Cdc42, which are also master regulators of the actin cytoskeleton and crucially involved in control of lamellipodia-dependent mesenchymal migration, were shown to inhibit bleb formation and amoeboid migration in various model systems^39^,^40^. Moreover, a discrete pool of active RhoA is reportedly essential for the local assembly of the contractile actomyosin ring and for cytokinesis^49^. In contrast, in numerous studies, it was shown that RhoB, RhoC, Rac1 and Cdc42 are not essential and dispensable for cytokinesis^49^,^50^,^51^,^52^. Moreover, our finding that the overexpression of human RhoA and the non-glycosylatable mutant RhoA Y34F resulted in the suppression of the toxic phenotype was in line with the view that RhoA plays a predominant role in Afp18-induced developmental defects. This hypothesis was strongly supported by the finding that the effector protein Afp18 directly targets RhoA by GlcNAcylation.

We determined the crystal structure of Afp18-GlcNAcylated RhoA and identified the covalent sugar modification at tyrosine-34. RhoA modified at this site revealed an opened switch I region, distinct from structures of RhoA bound to GDP^41^ or GTP^42^. It was shown that the switch I region of RhoA and especially tyrosine-34 is located in the interface of functional complexes of RhoA and its GEFs or its effectors^53^. We assume that steric constraints of the attached GlcNAc-residue results in the distorted conformation, which is incompatible with regulator and effector interaction. This assumption was underlined by the finding that the RhoGEF LARG, p50RhoGAP and the effectors ROCK and Rhotekin were unable to interact with Afp18-modified RhoA. Further experiments revealed that higher concentrations of Afp18 also GlcNAcylated Cdc42 and Rac *in vitro*. The modification of tyrosine-32 was verified by impaired glycosylation of site-directed mutants and in the case of Cdc42 by mass spectrometric analysis. Remarkably, we found that after the expression of Afp18 in target cells, which blocked RhoA functions and inhibited the interaction of the GTPase with its effectors, Rac1 and Cdc42 were still able to interact with their effector PAK. This suggests that *in vivo* Afp18 is specific for RhoA, a finding which is in agreement with our results on the toxic effects of Afp18 in zebrafish embryos and the time-resolved morphological effects on the actin cytoskeleton in zebrafish (ZF4) cells. However, the overexpression of RhoA or the non-glycosylatable mutant RhoA Y34F did not totally rescue Afp18-dependent developmental defects. Therefore, we cannot entirely exclude that Afp18^G^ modulates other Rho GTPases *in vivo* as well, which then may contribute to the developmental phenotype.

GlcNAcylation of tyrosine-34(32) of Rho GTPases was recently reported for PaTox, an exotoxin from entomopathogenic *Photorhabdus asymbiotica*^7^. In this study, PaTox-induced modification of RhoA was analysed by ^1^H-NMR spectroscopy. Our crystallization data extend these findings and show a conformation of the switch I region, which was not obvious from NMR data. Interestingly, both enzymes Afp18 and PaTox modify preferentially the active GTP-bound state of GTPases. While PaTox harbours an additional deamidase domain that deamidates heterotrimeric G proteins and is suggested to be involved in Rho activation, Afp18 possesses no typical deamidation domain. It remains to be clarified whether and how Afp18 causes activation of RhoA. Tyrosine-32(34) in Rho GTPases is also known to be modified by phosphorylation^54^,^55^, nitration^56^ and adenylylation (AMPylation)^57^ indicating a pivotal role of this residue in the endogenous regulation of Rho GTPase signalling. In contrast to well-known modifications of serine or threonine residues, glycosylation of a tyrosine residue has not been described in mechanistic detail.

Recently, several novel mono-*O*-glycosyltransferases were identified, which perform unusual amino-acid modifications in proteins, targeting arginine^44^,^45^, tryptophan^43^ and tyrosine^7^. Most of these enzymes derive from bacterial species and function as highly active bacterial toxins or effectors in eukaryotes. Site-directed mutagenesis of tyrosine-34 of RhoA to serine, threonine, tryptophan and arginine inhibited modification by the *Yersinia* glycosyltransferase showing that Afp18 is highly specific and accepts only tyrosine as an acceptor amino acid. Moreover, we analysed the stability of the covalent attached sugar by an *in vitro* deglycosylation assay with OGA that is the only known O-GlcNAcase in the eukaryotic host cytoplasm. In agreement with the crystal structure obtained, showing that GlcNAc is linked to RhoA in an α-anomeric configuration, OGA, which specifically cleaves β-glycosidic GlcNAc moieties, was not able to remove the sugar from RhoA. Thus, eukaryotic host cells seem not to be able to cope and revert these unusual post-translational modifications making Afp18 a highly efficient toxin and consequently, resistance against *Yersinia* Afp might not be possible by simply upregulating OGA expression in the prey cell.

Our knowledge about Afp prophage tail-like translocation machineries is still in its infancy. Recently, the Afp translocation apparatus of *Serratia entomophila*, the closest orthologue of the *Yersinia* Afp, has been described and first structural insights were obtained by negative stain electron tomography^20^,^23^. Afp from *Y. ruckeri* contains all genes required to build the contractile prophage tail injection apparatus and harbours the toxin effector Afp18, which is suggested to be caged inside the tail tube and injected into host cell cytoplasm^23^. Remarkably, from genome sequence analyses, a broad distribution of this system in prokaryotes including archaea has been proposed^21^. Afp-related systems are the *Photorhabdus luminescens* virulence cassette PVC and the phage tail-like R-type pyocins (also called bacteriocins), which share similarity with the type VI secretion system (T6SS). Effector proteins of phage tail-derived secretion systems have diverse functions both in competition with other prokaryote predators (antibactericidal activity) and in interaction with eukaryotic host cells. In contrast to common type VI secretion systems, the Afp system is probably released as preformed injection ‘torpedos' and is not associated to the bacterial donor cell^20^,^58^. Thereby, the system is extremely efficient; only 500 particles of *S. entomophila* Afp have been reported to kill the New Zealand grass grub (*Costelytra zealandica*) host^23^. Recent studies unravelled the mode of action of several T6SS effectors^59^,^60^,^61^,^62^. However, to date, the molecular mechanism of Afp effectors remained enigmatic. Thus, our findings that *Y. ruckeri* Afp18 harbours a glycosyltransferase, is the first report on a phage tail-derived pyocin effector targeting a specific regulatory protein (Rho GTPases) in the vertebrate host cell and might also contribute to human disease^32^. The results of our study on the effector component Afp18 might serve as a paragon for the fast growing number of phage-like pyocin producing pathogenic bacteria.

## Methods

### Materials and bacterial strains

DNA-modifying enzymes were obtained from Fermentas (St Leon-Rot, Germany), Phusion High-Fidelity DNA Polymerase from New England Biolabs (Ipswich, MA) and PfuUltra HF DNA Polymerase from Stratagene (Waldbronn, Germany). UDP-[^14^C]glucose and UDP-[^14^C]*N*-acetylglucosamine were from Biotrend (Cologne, Germany). UDP-[^14^C]galactose was from Perkin-Elmer Life Sciences (Rodgau, Germany). 2′(3′)-*O*-(*N*-methylanthraniloyl)-guanosine 5′-diphosphate (mant-GDP) and mant-guanosine 5′-[β, γ-imido]triphosphate (mant-GppNHp) were from Jena Bioscience (Jena, Germany). pET-28a vector was from Novagen (Madison, WI), pGEX4T1 and pGEX-4T3 were from GE Healthcare (Freiburg, Germany). *Yersinia ruckeri* (ATCC 29473) was from the German Collection of Microorganisms and Cell Cultures (DSMZ) and cultivated in CASO bouillon at 28 °C. *E. coli* TG1 was used for general cloning and protein expression of pGEX constructs. *E. coli* BL21 (DE3) CodonPlus (Stratagene) were used for protein expression of pET constructs. Toxin B was prepared from *Clostridium difficile* supernatants and purified by anion exchange chromatography^63^. The plasmids pGEX4T1-LARG (766–1,138) was kindly provided by Mohammad Reza Ahmadian (University Düsseldorf, Germany). All other reagents were of analytical grade and purchased from commercial sources. For all proteins used in this study the accession numbers and the species are listed in Supplementary Table 2.

### Antibodies

Anti-RhoA (3L74, dilution 1:500), anti-Cdc42 (Cat. No. 17–299, dilution 1:500), anti-Rac1 (23A8, dilution 1:2,500) and anti-GAPDH (6C5, dilution 1:20,000) antibodies were from Millipore (Schwalbach, Germany), anti-GFP (A10262, dilution 1:500), anti-chicken Alexa Fluor 488 (A11039, dilution 1:1,000), anti-rabbit Alexa Fluor 555 (A21430, dilution 1:1,000) antibodies were from Life Technologies (Darmstadt, Germany), and Horseradish peroxidase (HRP)-conjugated anti-MBP (Cat. No. E8038, dilution 1:5,000) was from New England Biolabs. Anti-GST (Cat. No. 27-4577-01, dilution 1:2,000) was from GE Healthcare, HRP-linked anti-mouse antibody from Rockland Immunochemicals (Limerick, PA) and HRP-conjugated anti-rabbit antibody from New England Biolabs.

### Cloning of genes for bacterial and zebrafish expression

The gene Afp18 (C4ULG3) and the fragments Afp18^G^ were amplified with Phusion DNA polymerase from the genomic DNA of *Y. ruckeri* (ATCC 29473) with oligonucleotide primers with additional restriction sites for BamHI and SalI (Supplementary Table 1). The genes were ligated into a predigested pET-28a vector with an introduced tobacco etch virus (TEV) protease cleavage site. QuikChange Kit (Stratagene, La Jolla, CA) in combination with PfuUltra HF DNA polymerase was used for the replacement of one to three nucleotides using the oligonucleotides shown in Supplementary Table 1. All sequences of corresponding plasmids were confirmed by sequencing (GATC Inc., Konstanz, Germany).

### Recombinant protein expression

*Escherichia coli* BL21* CodonPlus cells (Stratagene) transformed with the desired plasmid were grown in LB broth supplemented with the corresponding antibiotics on a shaker at 37 °C until A_600_=0.8. Protein expression from the pET28-based plasmids was induced by 1 mM isopropyl-β-D-thiogalactopyranoside (Roth, Karlsruhe, Germany) for 4–5 h at 22 °C, and pGEX-based expression was induced with 0.2 mM isopropyl-β-D-thiogalactopyranoside at 37 °C for 6 h. Bacterial cells were harvested by centrifugation at 6,000*g* for 15 min, resuspended in lysis buffer (50 mM Tris-HCl pH 7.4, 150 mM NaCl, 25 mM imidazole, 30 μg ml^−1^ DNase I, 1 mM β-mercaptoethanol and 1 mM phenylmethanesulfonylfluoride) and lysed by French press or ultra-sonic treatment. In the case of RhoA purification, 100 μM GDP was added to the lysis buffer. The cleared lysate was subjected to chromatography on a glutathione-Sepharose or nickel-equilibrated chelating Sepharose Fast Flow column, according to the manufacturer's instructions (GE Healthcare). Bound proteins were eluted with 10 mM reduced glutathione, 0.5 M imidazole, thrombin treatment or TEV protease treatment, depending on the construct used. Thrombin was removed by binding to benzamidine-Sepharose (GE Healthcare) and His-TEV by Ni^2+^-affinity chromatography. Further purification and removal of protein impurities or small molecular weight components as reduced glutathione, EDTA, labelled and unlabelled nucleotides or analogues was achieved by size-exclusion chromatography using Superdex 75 or Superdex 200 (each 10/300) columns. For crystallization, RhoA (amino acids 1–181) was expressed and purified as GST-tagged protein. After the removal of GST by thrombin treatment, RhoA was additionally purified by size-exclusion chromatography (Superdex 200, GE Healthcare).

### Cell culture

HeLa cells (ATCC CCL-2) were cultured in Dulbecco's modified Eagle's medium (DMEM) supplemented with 10% fetal calf serum (FCS), 1% non-essential amino acids, 4 mM penicillin, 4 mM streptomycin and 1% sodium pyruvate (Biochrom, Berlin, Germany). Cells were cultivated in a humidified atmosphere of 5% CO_2_ at 37 °C. Where necessary, the cells were starved overnight in DMEM without FCS. *Danio rerio* (ZF4) cells (ATCC CRL-2050) were cultivated in DMEM/F12 (Biochrom, Berlin, Germany) with 10% FCS, 4 mM penicillin, 4 mM streptomycin and Fungizone (Life Technologies) in 4% CO_2_ atmosphere at 28 °C. For intoxication of cells, GST-CNF1 (400 ng ml^−1^), native full-length *C. difficile* toxin B (TcdB, 1 ng ml^−1^) was applied into the medium or a combination of *B. anthracis* protective antigen (PA, 0.5 μg ml^−1^) and Afp18-fragments (370 ng ml^−1^) and incubated for 4 h if not otherwise stated. Transfection of cells was performed with peqFECT DNA (peqlab, Erlangen, Germany) as mentioned in the manufacturer instructions.

### Actin staining

ZF4 or HeLa cells grown on glass coverslips were washed with PBS, fixed with 4% formaldehyde in PBS and permeabilized with 0.15% (v/v) Triton X-100 in PBS for 10 min at room temperature. Subsequently, the cells were incubated with TRITC-conjugated phalloidin and washed again with PBS. Cells were embedded with Mowiol supplemented with 1,4-diazobicyclo[2.2.2]octane (Sigma) and analysed by fluorescence microscopy using an Axiophot system (Zeiss, Oberkochen, Germany) processed by using the Metamorph 7.0 software (Universal Imaging, Downingtown, PA).

### Injection of zebrafish embryos

Zebrafish (*Danio rerio*) were bred and maintained in our animal facility under standard conditions. For all microinjection methods, WT embryos obtained by single pair mating of 6–18-month-old male and female AB/TL strain zebrafish were used. Injections were performed at the one-cell stage except when indicated otherwise. Holding and breeding of zebrafish were in accord with the German laws for animal care under a permit obtained from the Regierungspräsidium Freiburg. Embryos were incubated in 0.3 × Danieau's medium or 1 × Danieau's with 1% v/v Penicillin (5,000 U ml^−1^)/Streptomycin (5,000 μg ml^−1^) solution (Gibco, Life Technologies) at 28.5 °C. Embryos, injected with recombinant proteins mRNA, or vector DNA were staged according to developmental progress of their non-injected littermates. For mRNA synthesis, SP6 mMessage mMachine Kit and T7 mMessage mMachine Ultra Kit (Ambion, Life Technologies) and the complementary DNAs of the following expression constructs in pCS2+ vector (Invitrogen, Life Technologies) were used: pET-28a(+) Afp18 and pET-28a(+) Afp18 NxN, linearized with NotI; pCS2+Afp18^G^ and pCS2+Afp18^G^ NxN linearized with NotI; pCS2+RHOA and pCS2+RHOA Y34F linearized with Acc65I. The original construct pEGFP-N1-Lifeact^64^ was subcloned into pCS2+ vector and linearized with NotI. The p13-pCS-H2B-mRFP^65^ construct was linearized with NotI. The pGI-GFP expression construct linearized with NotI was kindly provided by Gudrun Aspöck. We injected 100 pg *Lifeact-GFP*, 100 pg *H2B-mRFP*, 50 pg *GFP*, 50 pg of *RHOA* or *RHOA Y34F*, 0.5 pg of *Afp18*^*G*^ or *Afp18*^*G*^ *NxN* mRNA constructs per embryo at one-cell stage unless otherwise indicated. Functionality and quality of mRNA was checked by *in vitro* translation using Transcend Non-radioactive Translation Detection Systems (Promega Corporation, Madison) of the corresponding mRNA, followed by western blotting (Supplementary Fig. 5b). Afp18^G^ activity of *in vitro* translated protein was additionally validated by an *in vitro* glycosylation reaction with recombinant RhoA. For mosaic labelling, plasmid vectors of the following constructs were injected in one blastomere at 16-cell stage: pCS2+ RHOA, pEGFP-C1-Afp18^G^ and pEGFP-C1-Afp18^G^ NxN.

### Microscopy and imaging acquisition

For overviews of embryos in 0.3 × or 1 × Danieau's, a MZ APO stereo microscope (Leica, Wetzlar, Germany) with an AxioCam MRc camera and AxioVS40 V4.8.0.0 software (Zeiss, Jena, Germany) or fluorescent stereo microscope (Leica M165 FC and Leica Application Software (Leica, Mannheim, Germany) was used. For wide-field microscopy, an Axio Examiner.D1 (objective. Plan-neofluar 20 × 1.0) and Zen 2012 (blue edition) software was used (Zeiss, Jena, Germany). For confocal microscopy, an Axio Observer.Z1 spinning disc (objective: LD C-Apochromat × 40/1.1 W) and an Axio Imager Z1 (objective Plan-Apochromat × 63/1.2 W) for single-plane images was used. Images were acquired with ZEN 2012 software (Zeiss, Jena, Germany). Embryos were mounted in 0.8% low melting temperature agarose (Biozyme Sieve GP Agarose, Biozym Scientific, Germany) dissolved in 0.3 × Danieau's or for fixed samples in PBS. Time-lapse movies of *z* stacks were acquired with the Axio Observer.Z1 spinning disc microscope. The confocal parameters of the time series were: average *z*-stack depth about 30 μm (2.5 μm per slice) with 3-min intervals for a total of 1 h unless otherwise noted. Time series were analysed and processed with Zen 2012 (blue edition) software. Figure panels were generated using Adobe Photoshop CS4 Extended. When image adjustments were necessary, all images representing control and experimental samples were adjusted in an identical manner by using the Photoshop ‘levels' tool to spread the intensity values linearly to fill the 256 values of the RGB spectrum. Images show cells or embryos representative for each experimental condition.

### Immunodetection

Embryos were fixed with 4% paraformaldehyde in PBS for 2 h at room temperature. After washing with PBST (PBS, 0.5% Triton X-100), the embryos were dechorionated manually. Embryos were dehydrated with methanol overnight at −20 °C. Rehydrated embryos were blocked in PBST, 5% goat serum, 1% BSA for 2 h and incubated with primary (anti-GFP and anti-Rho) antibodies in blocking solution overnight. Embryos were washed 6 h with PBST followed by the secondary antibody (anti-chicken Alexa Fluor 488 and anti-rabbit Alexa Fluor 555) incubation in blocking solution. The embryos were finally washed 5 × in PBST before imaging.

### Hydrolysis of UDP-sugars by Afp18^G^

UDP-sugar hydrolysis was measured as described earlier^66^. Afp18^G^ (100 nM) was incubated with 10 μM UDP-[^14^C]-sugars in a buffer, containing 50 mM Hepes pH 7.5, 2 mM MgCl_2_ and 1 mM MnCl_2_. Total volume was 10 μl. After incubation for 15 min at 30 °C, samples of 800 nl were taken and subjected to PEI (polyethylenimine)-cellulose thin-layer chromatography (Merck, Darmstadt, Germany) with 0.2 mM LiCl as mobile phase to separate the hydrolysed sugar from intact UDP-sugar. The plates were dried and analysed by PhosphorImager analysis. Quantification was carried out with ImageQuant (GE Healthcare).

### Glycosylation reaction

Recombinant Afp^G^ (10 nM if not otherwise stated) was incubated with 10 μM UDP-[^14^C]*N*-acetylglucosamine in a buffer, containing 50 mM HEPES pH 7.4, 2 mM MgCl_2_ and 1 mM MnCl_2_ for 20 min at 30 °C in the presence of recombinant GST-tagged GTP-binding proteins (2 μM) or cell lysate. Total volume was 20 μl. Labelled proteins were analysed by SDS–polyacrylamide gel electrophoresis (SDS–PAGE) and phosphorimaging. Uncropped gels and autoradiographs from the corresponding figures of the main text are presented in Supplementary Fig. 6. For quantitative modification, GST-RhoA bound to glutathione-Sepharose beads were modified with Afp18^G^ (10 nM) and UDP-GlcNAc (100 μM). Beads were extensively washed with glycosylation buffer and RhoA eluted by thrombin cleavage in buffer C (10 mM TEA pH 7.5, 150 mM NaCl and 2.5 mM MgCl_2_). Thrombin was removed by incubation with benzamidine-Sepharose. Complete glycosylation was confirmed by a second *in vitro* glycosylation reaction. All glycosylation reactions were repeated three times.

### GlcNAz labelling

Recombinant RhoA (6 μg), TAB1 (1 μg) or cell lysate (130 μg) was incubated with 4 μM uridine diphosphate *N*-azidoacetylglucosamine (UDP-GlcNAz) in the presence of Afp18^G^ or Afp18^G^ NxN (each 24 nM) in a buffer containing 10 mM Hepes, pH 7.5, 2 mM MgCl_2_, 1 mM MnCl_2_ and 0.1 mg ml^−1^ BSA for 60 min at 30 °C. Detection of GlcNAz-modified proteins was performed by click chemistry reaction with biotin alkyne according to the manufacturer's instructions (Click-iT Biotin Protein Analysis Detection Kit, Molecular Probes, Darmstadt, Germany) and western blotting using HRP-coupled streptavidin (dilution 1:10,000, Cell Signaling, Danvers, MA).

### Deglycosylation reaction

Recombinant RhoA (6 μg), TAB1 (1 μg) or Rho GTPases of cell lysate (130 μg) were labelled with either radiolabeled UDP-[^14^C]GlcNAc or UDP-GlcNAz in a glycosylation reaction with Afp18^G^ (24 nM) or human *O*-glycosyltransferase (OGT, 0.5 μM) as described above. The reaction volume was 20 μl. Subsequently, 0.5 μM recombinant human OGA or *Clostridium perfringens* CpNagJ were added to the reaction mixture for 60 min at 37 °C. Remaining glycosides were detected by radiography or biotin alkyne click chemistry with western blotting using HRP-coupled streptavidin, respectively.

### Mass spectrometry

Glycosylation of RhoA, Rac1 and Cdc42 (5 μg each) was performed with Afp18^G^ (100 nM) in the presence of UDP-GlcNAc (1 mM) for 30 min at 30 °C in glycosylation buffer. After SDS–PAGE, excised gel bands were destained with 30% acetonitrile (ACN), shrunk with 100% ACN and dried in a vacuum concentrator. Subsequently, protein digestion with thermolysin was performed overnight at 37 °C in 0.1 M NH_4_HCO_3_ (pH 8). Approximately 0.1 μg of protease per gel band was used. Peptides were extracted from the gel matrix with 5% formic acid and subsequently analysed by LC–MS on a Q-TOF mass spectrometer (Agilent 6520, Agilent Technologies) coupled to a 1200 Agilent nanoflow system via a HPLC-Chip cube electrospray ionization interface. Peptides were separated on a HPLC-Chip with an analytical column of 75 μm i.d. and 150 mm length and a 40-nl trap column, both packed with Zorbax 300SB C-18 (5 μm particle size). Starting with 3% ACN, a linear gradient with 1% per min at a flow rate of 300 nl min^−1^ was applied. The Q-TOF instrument was operated in the 2-GHz extended dynamic range mode and MS spectra were acquired in the mass-to-charge (*m/z*) range between 50 and 3,000. For MS–MS analyses, the instrument was operated in the data-dependent acquisition mode. After a survey MS scan (four spectra s^−1^), a maximum of three multiple charged peptides were consecutively selected for MS–MS experiments (two spectra s^−1^). Internal mass calibration was applied. Mascot Distiller 2.4.2 was used for raw data processing and for generating peak lists, essentially with standard settings for the Agilent Q-TOF instrument. Mascot Server 2.3 was used for searches in the SwissProt protein database with the following parameters—peptide mass tolerance, 50 p.p.m.; MS–MS mass tolerance, 0.05 Da; enzyme, no specificity; variable modifications, Carbamidomethyl (C), Gln-> pyroGlu (N-term. Q), oxidation (M) and HexNAc (STY).

### Effector pull-down assay

The Rho-binding region of Rhotekin (amino acids 1–90) and the CRIB-domain of PAK (amino acids 56–272) were expressed as GST-fusion protein in *E. coli* BL21 and purified by affinity chromatography with glutathione-Sepharose beads (GE Healthcare). The Rho-binding region of Rho kinase α (ROCKII) was expressed as maltose-binding protein (MBP)-fusion and purified with amylose-resin (New England Biolabs). HeLa or ZF4 cells were treated for 4 h with or without GST-CNF1 (400 ng ml^−1^) to activate Rho GTPases and a combination of PA (0.5 μg ml^−1^) and *Yersinia* Afp18 glucosyltransferase fragments (100 ng ml^−1^) or *C. difficile* toxin B (TcdB, 1 ng ml^−1^). After stimulation of the cells with serum (10% FCS) for 10 min, cells were lysed in ice-cold buffer A (50 mM Tris pH 7.4, 100 mM NaCl, 1% NP-40, 10% glycerol, 2 mM MgCl_2_ and 1 mM phenylmethanesulfonylfluoride) and cellular debris were removed by centrifugation (3 min, 17,000 *g*). A fraction of the cleared lysates (50 μg of total protein) was analysed by immunoblotting to detect total amounts of the respective GTPases. The lysates were incubated for 20 min at 4 °C with MBP-ROCKII, GST-Rhotekin or GST-PAK immobilized on beads. The beads were precipitated and washed with buffer A. Finally, the proteins were subjected to SDS–PAGE and transferred onto polyvinylidene difluoride membranes and GTPases detected by using specific antibodies and HRP-linked secondary antibodies. Uncropped western blots from the corresponding figures of the main text are presented in Supplementary Fig. 6.

### Mant-GDP- and mant-GppNHp-binding

The measurements were carried out on a LS50B spectrofluorometer from Perkin-Elmer at 37 °C. The amount of nucleotide (GDP/GTP) bound to RhoA was determined by ion-pair reversed-phase liquid chromatography using a C-18 RP column (LiChrosorb 5 μm, 250 × 4 mm) using an Agilent 1100 HPLC system with a calibrated detector system (absorbance of guanine at 252.4 nm). The separation of the nucleotides was performed at ambient temperature with a flow rate of 1 ml min^−1^ with an isocratic phosphate buffer (50 mM, pH 6.5) containing 10 mM tetra-butylammoniumbromide and 7.5% acetonitrile. For nucleotide exchange experiments, 98.6±1.3% of RhoA was preloaded with GDP. The nucleotide exchange of RhoA was measured over the time by following the increase in fluorescence intensity at *λ*_em_=444 nm (*λ*_ex_=360 nm) of mant-nucleotides on binding to RhoA. Mant-GDP or mant-GppNHp (each 1 μM) was incubated with RhoA (0.5 μM) in degased buffer C (10 mM TEA, pH 7.5, 150 mM NaCl and 2.5 mM MgCl_2_). The samples were excited in a cycle of 1 min for 1 s.

### Guanine nucleotide exchange reaction measurements

WT RhoA and Afp18^G^-preglycosylated RhoA (each 0.5 μM) were incubated with mant-GDP or mant-GppNHp (each 1 μM) in degased buffer C at 20 °C in the absence or presence of 100 nM LARG and incubated for the indicated time periods. Fluorescence was measured with a LS50B spectrofluorometer at *λ*_em_=460 nm (*λ*_ex_=360). Each experiment was repeated at least twice.

### Assay for GAP-stimulated GTPase activity

RhoA (3.6 μM) was incubated with Afp18^G^ (170 nM) in the absence (RhoA control) or presence UDP-GlcNAc (330 μM) (RhoA-GlcNAc). Subsequently, RhoA was loaded with [γ-^32^P]GTP (20 μM) for 5 min at 37 °C in loading buffer (50 mM Tris-HCl pH 7.5, 10 mM EDTA and 2 mM DTT). MgCl_2_ (25 mM, final concentration) and unlabelled GTP (2 mM, final concentration) were added. GAP-stimulated GTPase activity was measured at 25 °C by the addition of the 30 kDa active fragment of p50RhoGAP (550 nM, final concentration). At the indicated time points, proteins were collected by filtration through wet 0.22 μm nitrocellulose filter discs. The filters were washed three times with ice-cold buffer A (50 mM Tris-HCl, pH 7.5, 50 mM NaCl and 5 mM MgCl_2_), and protein-bound [γ-^32^P]GTP was quantified by liquid scintillation counting.

### RhoA-GlcNAc crystallization

A truncated version of RhoA covering amino acids 1–181 was expressed from a pGEX-based plasmid as GST-fusion protein. The tag was removed by thrombin cleavage in a buffer containing 50 mM Tris-HCl pH 7.5, 100 mM NaCl, 5 mM MgCl_2_, 5 mM dithiotreitol and 10% glycerol. After removal of thrombin by benzamidine-sepharose beads, RhoA (15 mg ml^−1^) was glycosylated by incubation with His-Afp18^G^ (170 nM) in the presence of 1 mM UDP-GlcNAc at 30 °C for 30 min. Quantitative glycosylation was proven by a second glycosylation with Afp18^G^ using radiolabelled UDP-[^14^C]GlcNAc. After the removal of His-Afp18^G^ by Ni^2+^ affinity chromatography, RhoA-GlcNAc was purified by size-exclusion chromatography Superdex 200 (10/300) in a buffer containing 10 mM HEPES pH 7.4, 50 mM NaCl and 1 mM MgCl_2_, concentrated to 7.7 mg ml^−1^ and crystallized in a 1:1 mixture with reservoir solution (0.1 M sodium acetate, pH 5 and 1.5 M ammonium sulfate). Crystals were obtained at 20 °C after 6 days with the sitting-drop vapour diffusion method and measured after cryoprotection with 20% glycerol in mother liquor.

### Structure determination

Diffraction data were collected at 100 K with a Rigaku M007HF X-ray generator and a Saturn 944HG+ CCD detector. The wavelength was 1.54 Å. Data were processed with HKL3000R. The initial phases were calculated by molecular replacement using Molrep from the CCP4 software suite^67^ with RhoA (PDB code 1pdf) as a search model using the reflections from 50 to 3.5 Å. The structure was further refined by rigid body and iterative restraint refinement with the software Refmac5 (ref. 68) and model building in COOT^69^. Structural data are summarized in Table 1. The electron density of the GlcNAc moiety, GDP and a sulfate were clearly observed in the unbiased F_o_−F_c_ map, but no Mg^2+^ ion was found. Electron density of the side chains from amino acid Glu32, Arg128, and Glu142 were poorly defined; therefore, amino acids were modelled as alanines. A crystallographic information file with the refinement restraints of the alpha-GlcNAc-Tyr bond was created in JLigand^70^. Structure images were prepared using PyMOL (http://www.pymol.org/).

### Statistical analysis

Analysis of variance was performed using GraphPad PRISM. Kruskal–Wallis and Mann–Whitney *U*-tests were applied. Student's *t*-test was applied for pairwise statistical comparison. Fisher's exact test was applied for statistical analysis of the data set in Fig. 2b, using VassarStats (http://vassarstats.net/).

## Additional information

**Accession codes:** Atomic coordinates and structure factors for the reported crystal structure are deposited at the RCSB data bank under accession code 5A0F.

**How to cite this article:** Jank, T. *et al.* Tyrosine glycosylation of Rho by *Yersinia* toxin impairs blastomere cell behaviour in zebrafish embryos. *Nat. Commun.* 6:7807 doi: 10.1038/ncomms8807 (2015).

## Supplementary Material

Supplementary Figures and Tables Supplementary Figures 1-6 and Supplementary Tables 1-2

Supplementary Movie 1 Time lapse movie of blastomeres expressing Afp18G NxN and Afp18G. Embryos were labeled with Lifeact-GFP and H2B-dsRed. Time series of confocal stacks (total depth = 5 ȝm) of the blastoderm were recorded at 2.1 min intervals (total duration = 60 min) upper row shows DIC image series, lower row epifluorescent image series. Asterisk marks an exemplified dividing cell failing in cytokinesis. Animal view, scale bar, 20 μm. (Related to Figure 4a).

Supplementary Movie 2 Time lapse movie of blastomere membrane protrusions of Afp18G NxN and Afp18G injected embryos. Embryos were labeled with Lifeact-GFP. Widefield images of the blastoderm were recorded at 10 s intervals (total duration = 5 min). Upper row shows DIC image series, lower row the corresponding epifluorescent image series. Asterisk marks bleb formation, tick shows lamellipodia formation. Animal view, scale bar, 10 μm. (Related to Figure 5).

Supplementary Movie 3 Effects of Afp18G on the actin cytoskeleton. Time lapse movie of GFP-actin expressing ZF4 cells intoxicated with recombinant Afp18G NxN and Afp18G in combination with PA as delivery system. (Related to Figure 3c)

## Figures and Tables

**Table 1:** Data collection, phasing and refinement statistics for RhoA^Y34(GlcNAc)^.

	**RhoA**^Y34(GlcNAc)^
*Data collection*	
Space group	P4_3_ 2_1_ 2
Cell dimensions	
*a*, *b*, *c* (Å)	91.39, 91.39, 56.62
*α*, *β*, *γ* (°)	90, 90, 90
Resolution (Å)	50–2.0 (2.07–2.00)
*R*_merge_	0.108 (0.319)
*I*/σ*I*	21.3 (5.5)
Completeness (%)	100.0 (100.0)
Redundancy	5.8 (5.6)
	
*Refinement*	
Resolution (Å)	50–2.0
No. of reflections	185,873
No. of unique reflections	31,941
*R*_work_/*R*_free_	0.206/ 0.213
No. of atoms	
Protein	1,399
GlcNAc	14
GDP	28
Sulfate	5
Water	80
*B*-factors	
Protein	19.73
GlcNAc	18.65
GDP	26.32
Sulfate	24.75
Water	22.75
r.m.s deviations	
Bond lengths (Å)	0.013
Bond angles (°)	1.67

Abbreviation: r.m.s., root mean squared.

For each data set one crystal was measured. *Values in parentheses are for highest-resolution shell.
