# Supplementary material for: Tyrosine glycosylation of Rho by Yersinia toxin impairs blastomere cell behaviour in zebrafish embryos
Source: Nat Commun. 2015 Jul 20;6:7807. doi: 10.1038/ncomms8807 (PMC4518317; doi:10.1038/ncomms8807)
Supplement: Supplementary Figures and Tables — Supplementary Figures 1-6 and Supplementary Tables 1-2 [file ncomms8807-s1.pdf]

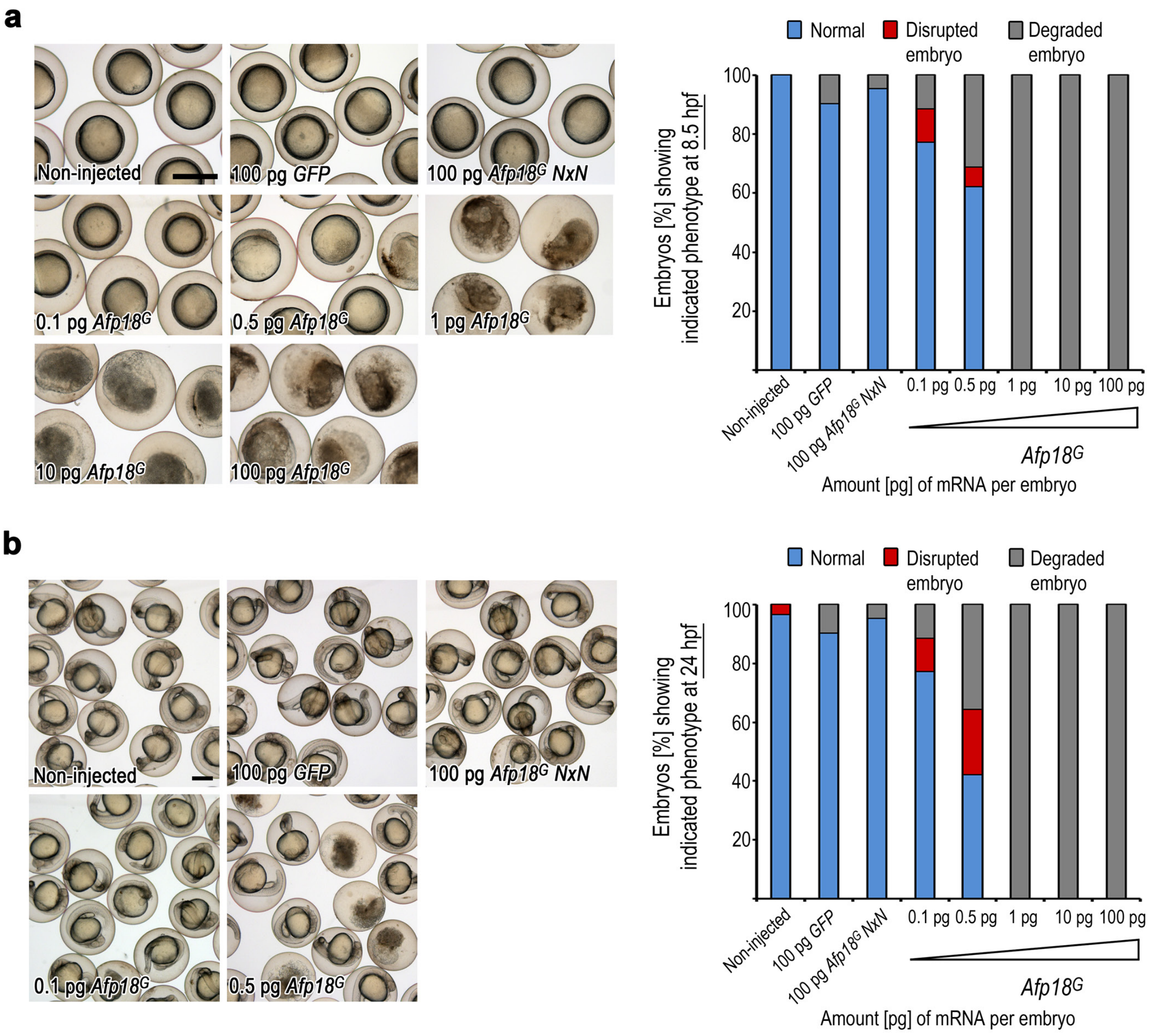

Supplementary Figure 1. Concentration dependent effect of Afp18^{G} on zebrafish embryos.

(**a**) Live images of non-injected, *GFP* mRNA (100 pg/embryo), *Afp18^{G} NxN* mRNA (100 pg/embryo), or different amounts (0.1 pg – 100 pg/embryo) of *Afp18^{G}* mRNA injected embryos at late gastrula (8.5 hpf). Scale bar, 500 µm. Graph gives percentage of embryos showing disintegrated embryos, disrupted blastoderms, or normal phenotypes. By late gastrulation, even embryos injected with small amounts only of *Afp18^{G}* mRNA frequently disintegrate (non-injected, *n*=41; *GFP* mRNA, *n*=37; *Afp18^{G} NxN* mRNA, *n*=52; 0.1 pg *Afp18^{G}*, *n*=35; 0.5 pg *Afp18^{G}*, *n*=45; 1 pg *Afp18^{G}*, *n*=60; 10 pg *Afp18^{G}*, *n*=62; 100 pg *Afp18^{G}*, *n*=60).

(**b**) Live images of non-injected, *GFP* mRNA (100 pg/embryo), *Afp18^{G} NxN* mRNA (100 pg/embryo), or different amounts (0.1 pg – 100 pg/embryo) of *Afp18^{G}* mRNA injected embryos at 24 hpf. Scale bar, 500 µm. Graph gives percentage of embryos which died and disintegrated, or show disrupted development with impaired body formations, or embryos which develop normally (non-injected, *n*=41; *GFP* mRNA, *n*=37; *Afp18^{G} NxN* mRNA, *n*=52; 0.1 pg *Afp18^{G}*, *n*=35; 0.5 pg *Afp18^{G}*, *n*=45; 1 pg *Afp18^{G}*, *n*=60; 10 pg *Afp18^{G}*, *n*=62; 100 pg *Afp18^{G}*, *n*=60).

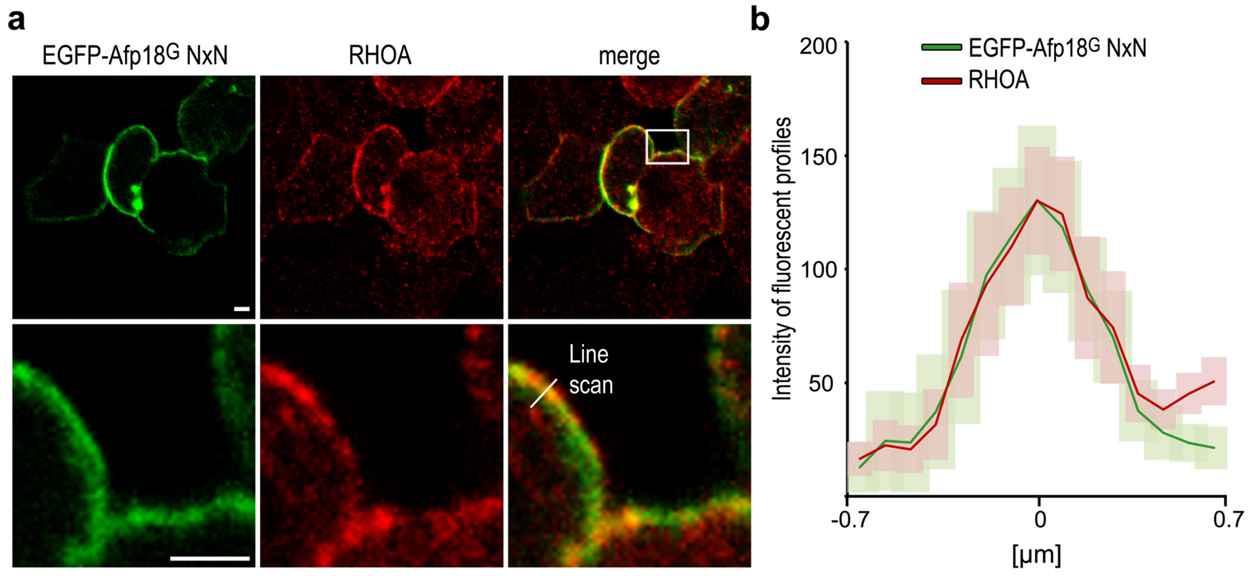

Supplementary Figure 2. Afp18^{G} NxN colocalizes with RHOA at the cell membrane.

(**a**) Confocal images (single plane) of double immunofluorescence against EGFP-Afp18^{G} NxN and RHOA at 60% epiboly. EGFP-Afp18^{G} NxN and RHOA vector DNA were injected in single blastomeres at 16-cell stage. Upper row: single channel images and merged image of representative mosaic labeled blastomeres. Lower row: Magnification of region depicted as white rectangle of the upper row. EGFP-Afp18^{G} NxN is targeted to the membrane and co-localizes with RHOA. Scale bar 2 µm.

(**b**) Corresponding graph shows evaluation of co-localization. Line scans were recorded along the same regions of blastomere membranes (representatively depicted by white line). Average fluorescent profiles of the line scans illustrate co-localization of Afp18^{G} NxN with RHOA at the membrane (n = 8 line scans of different blastomeres and embryos). Zero represents the manually defined center point of the membrane, (-) values correspond to distance to the outside, (+) values to the distance to the inside of the labelled blastomere.

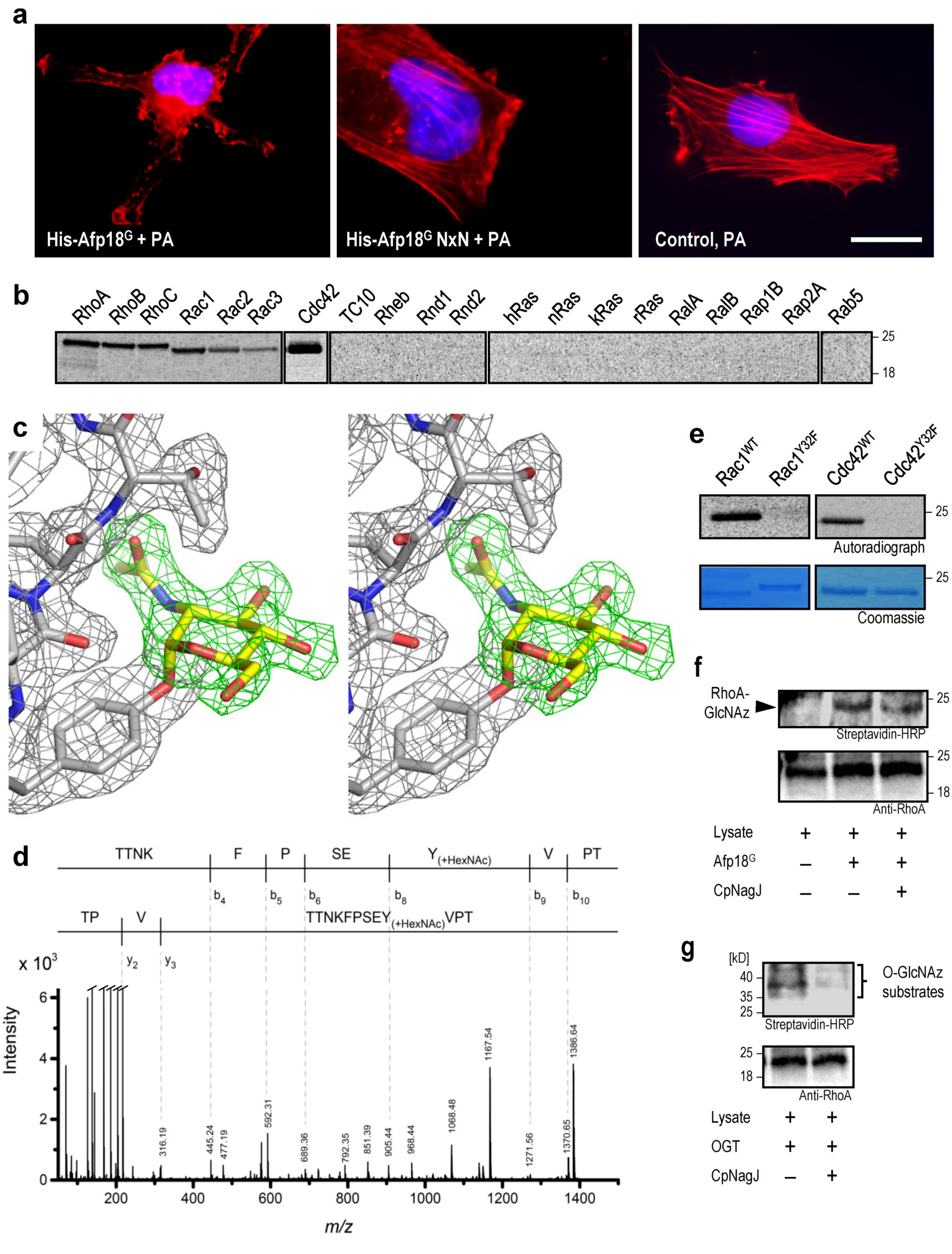

Supplementary Figure 3. Consequences of Afp18^{G} catalyzed tyrosine GlcNAcylation.

(**a**) HeLa cells were intoxicated with 6xHis-tagged Afp18^{G} and Afp18^{G} NxN in combination with anthrax protective antigen (PA) as translocation system or PA alone as control. Actin cytoskeleton was stained with TRITC-phalloidin and the nucleus was stained with DAPI. Scale bar, 10 µm.

(**b**) Autoradiogram of SDS-PAGE to assess substrate specificity of Afp18. Rho, Ras, and Rab family proteins were *in vitro* glycosylated by Afp18^{G} with UDP-[^{14}C]GlcNAc.

(**c**) Stereo representation of the α-GlcNAc modified switch I tyrosine-34 in the crystal structure of RhoA-GlcNAc. Electron density maps for the *N*-acteylglucosaminyl moiety (F_o-F_c omit map, *green mesh*) was contoured at 2.5 σ and for the protein (2F_o-F_c, *grey mesh*) at 1 σ. Protein is depicted with atom colors and grey carbons, the GlcNAc moiety with yellow carbons.

(**d**) Tandem mass spectrometric analysis of Afp18-catalyzed GlcNAcylation of Cdc42. MS-MS spectrum of the GlcNAc-modified peptide $_{24}$TTNKFPSEYVPT$_{35}$ of Afp18^{G}-modified Cdc42. Sequence-specific fragment *b*-type and *y*-type ions are annotated. Switch I Y32 residue in Cdc42 was identified as acceptor amino acid for GlcNAc. *m/z*, mass-to-charge.

(**e**) Autoradiograms and Coomassie stainings of Afp18^{G}-catalyzed *in vitro* ^{14}C-GlcNAcylation of wild-type (WT) Rac1 and Cdc42 and the indicated mutants.

(**f**, **g**) β-*O*-GlcNAcase of *Clostridium perfringens* (CpNagJ) is not able to deglycosylate RhoA. HeLa cell lysate was modified with UDP-GlcNAz by Afp18^{G} (f) or *O*-GlcNAc-transferase (OGT) (g). Subsequently, lysate was incubated with recombinant CpNagJ and analyzed by click chemistry and Western blotting. Western blot analysis of RhoA is shown as input control.

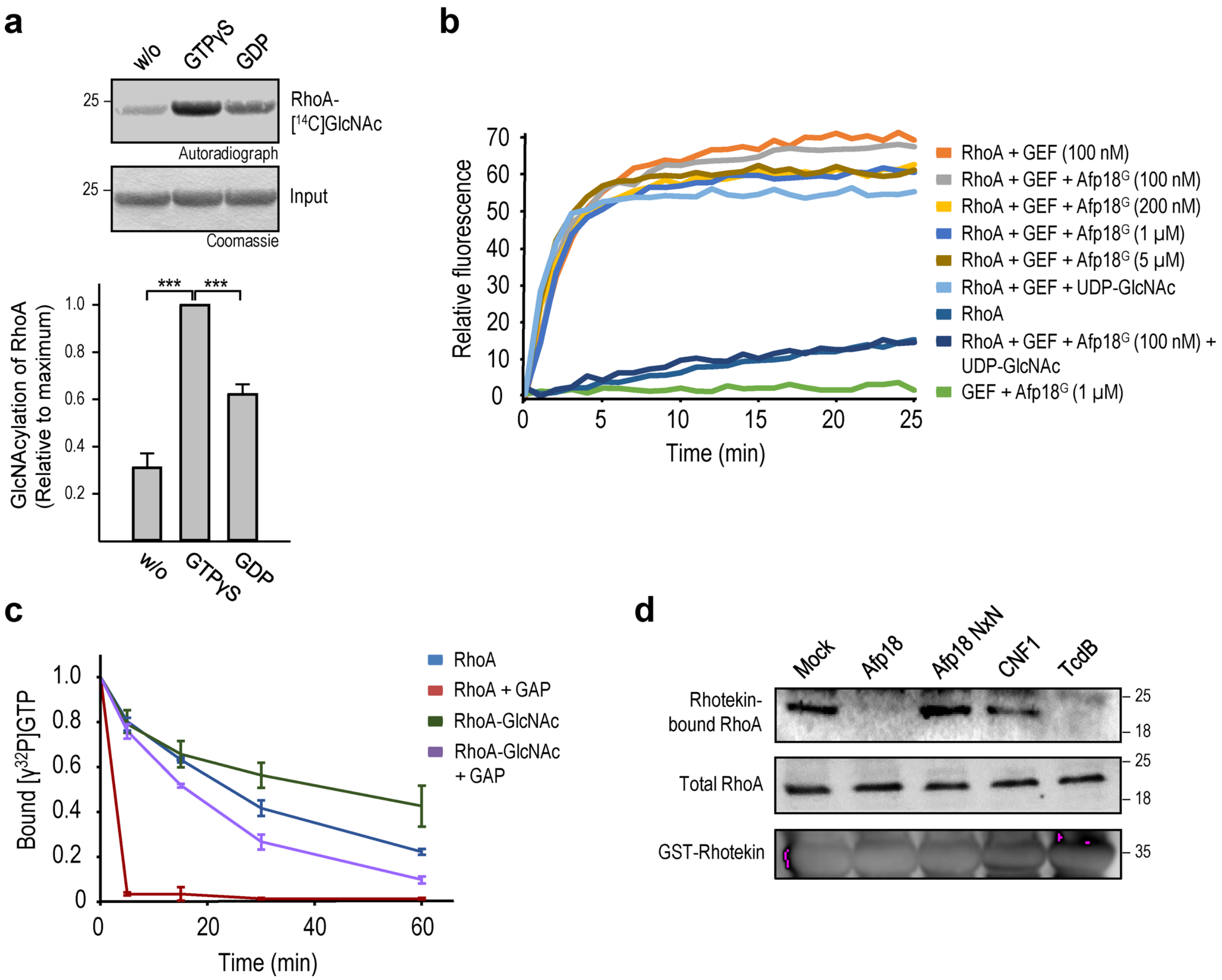

Supplementary Figure 4. Afp18-mediated glycosylation of RhoA is GTP-state specific, impairs Rhotekin effector interaction, GAP-stimulated GTPase function, and GEF-mediated nucleotide exchange.

(**a**) The GTP-bound conformation of RhoA is the preferred substrate for GlcNAcylation by Afp18. Autoradiogram and Coomassie staining of Afp18^{G}-catalyzed *in vitro* ^{14}C-GlcNAcylation of RhoA without (*w/o*) nucleotide or RhoA preloaded with GTPγS or GDP (*top panel*). Graph (*lower panel*) shows the quantification of three biological replicates. Data are presented as means ± s.e.m.. Asterisks represent significant *P*-values, ***$P < 0.001$, Student's *t*-test.

(**b**) Time course of GEF (LARG)-catalyzed mant-GDP nucleotide exchange of RhoA (0.5 μM) in the presence of increasing concentration of Afp18^{G} (100 nM – 5 μM) without or in the presence of UDP-GlcNAc (2 μM). Controls without GEF, Afp18^{G}, and RhoA are shown.

(**c**) Afp18^{G}-mediated GlcNAcylation of RhoA inhibits the function of RhoGAP. Time course of [γ-^{32}P]GTP hydrolysis by wild-type RhoA and GlcNAcylated RhoA in the presence and absence of p50Rho-GAP. Non-hydrolyzed [γ-^{32}P]GTP bound to RhoA was determined. Data are relative to initial loading (mean ± s.d., $n = 3$ technical replicates).

(**d**) Western blot analysis of RhoA pulldown experiments with Rho effector Rhotekin from HeLa cells treated with Afp18^{G} (plus PA for delivery), Afp18^{G} NxN (plus PA for delivery), cytotoxic necrotizing factor (CNF1), and *C. difficile* toxin B (TcdB). After Rho GTPase activation with fetal calf serum, active RhoA was pulled down with Rhotekin-beads. Bound RhoA was detected by anti-RhoA antibody. Immunoblot of total RhoA is the loading control.

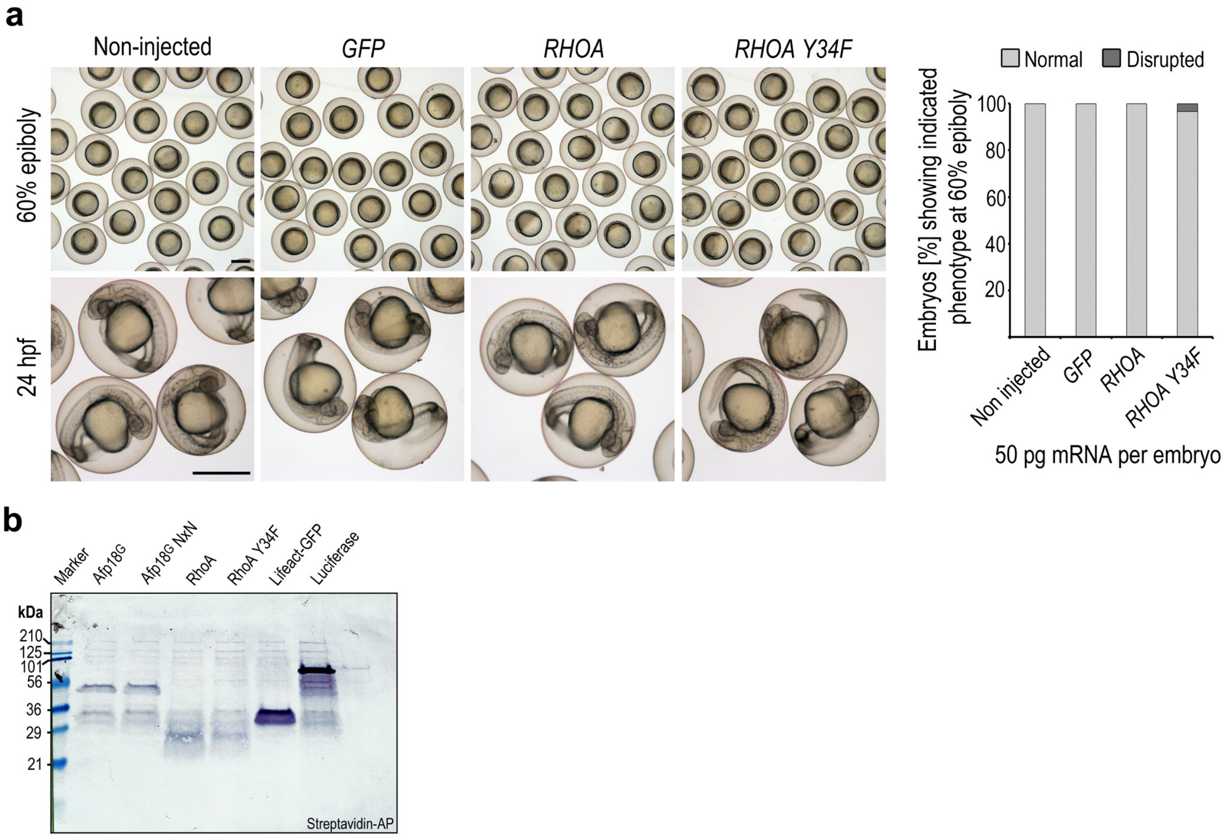

Supplementary Figure 5. Overexpression of RhoA Y34F and RhoA WT does not influence zebrafish development.

(**a**) Live images of non-injected, *GFP*, *RHOA, and RHOA Y34F* mRNA (50 pg/embryo each) injected embryos at 60% epiboly (7 hpf) and 24 hpf. *GFP*, *RHOA*, and *RHOA Y34F* injected embryos developed indistinguishable from non-injected controls. Scale bars, 500 μm. Quantification is shown in corresponding graph (non-injected *n* = 117 embryos; *GFP* mRNA *n* = 85; *RHOA* mRNA *n* = 114; RHOA Y34F mRNA *n* = 61).

(**b**) Western blot analysis of proteins *in vitro* translated from mRNAs as indicated. We used A*fp18*^{G}, *Afp18*^{G} *NxN*, *RHOA, RHOA Y34F*, and *Lifeact-GFP* expression constructs to synthesize mRNAs for *in vitro* translation. Luciferase mRNA, provided by Promega, served as loading control according the manufacturers manual of Transcend^{TM} Non-radioactive Translation Detection System (Promega). The electrophoretic mobility of proteins correlates with predicted molecular masses (Afp18^{G} 40 kDa, RhoA 22 kDa, Lifeact-GFP 33 kDa, Luciferase 63 kDa); the minor non-specific bands (200, 80, 32, and 17 kDa) also occur in control reaction and thus derive from mRNAs still active in the translation mix.

Fig. 6c

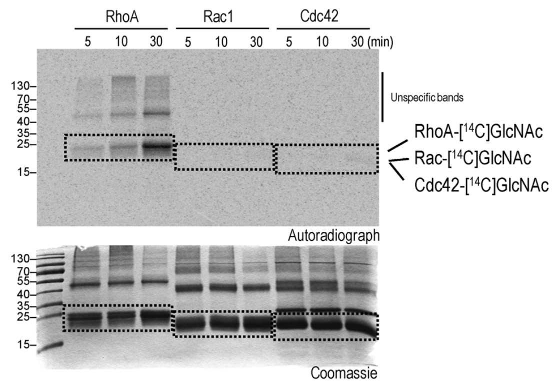

Fig. 6e

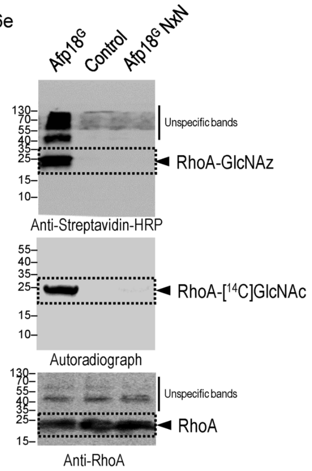

Fig. 7d

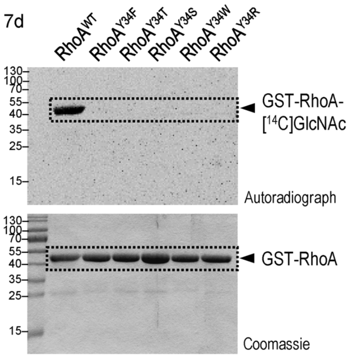

Fig. 7e

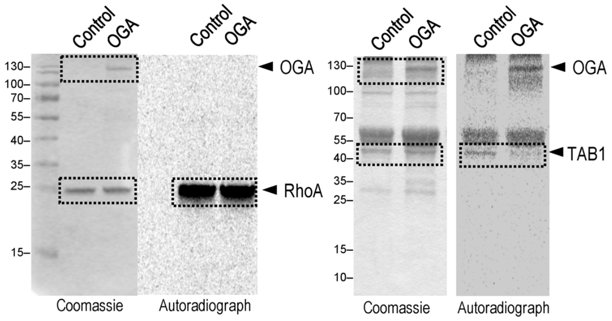

Fig. 8c

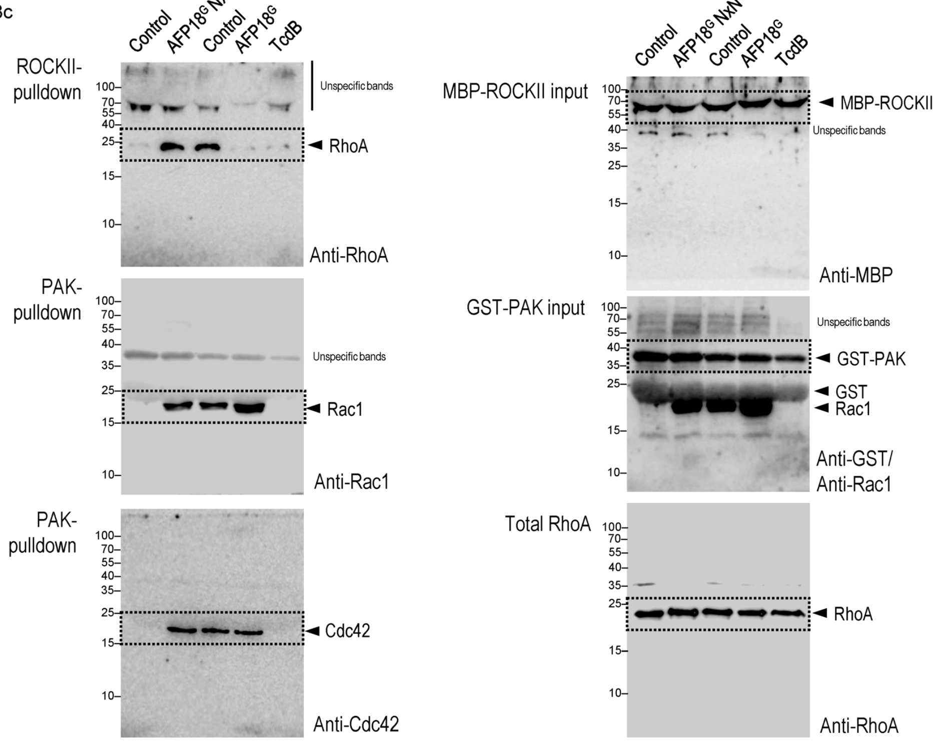

Supplementary Figure 6. Full length images of SDS-PAGE gels, immunoblots and autoradiographs from the corresponding figures of the main text including molecular weight markers in [kDa]. Boxes represent areas used in the figures.

Supplementary Table 1. Oligonucleotides used in this study

#	*Name*	*Sequence*
oligoTJ110	Afp18 rv Sal I	GACGCGTCGACTTAGCTCAGGCCTGAGCTTTGAGTGCTGTTAC
oligoTJ115	Afp18 fw BamH1	GACCTAGGATCCATGCCTTACTTTAATAAATCGAAGAAAAATG
oligoTJ164	Afp18 1771fw BamHI	GACCTAGGATCCATGGTGTTTGACTCTGCAAATACCAATCG
oligoTJ169	AFP18 rv XhoI	CTCGAGTTAGCTCAGGCCTGAGCTTTGAGTGC

Oligonucleotides for site-directed mutagenesis

#	*Name*	*Sequence*
oligoTJ65	RhoA Y34F fw	CAGTTCCCAGAGGTGTTTGTGCCCACAGTG
oligoTJ66	RhoA Y34F rv	CACTGTGGGCACAAACACCTCTGGGAACTG
oligoTJ88	RhoA Y34T fw	CAGTTCCCAGAGGTGACTGTGCCCACAGTG
oligoTJ89	RhoA Y34T rv	CACTGTGGGCACAGTCACCTCTGGGAACTG
oligoTJ90	RhoA Y34S fw	CAGTTCCCAGAGGTGTCTGTGCCCACAGTG
oligoTJ91	RhoA Y34S rv	CACTGTGGGCACAGACACCTCTGGGAACTG
oligoTJ142	RhoA Y34R fw	CAGTTCCCAGAGGTGCGGGTGCCCACAGTG
oligoTJ143	RhoA Y34R rv	CACTGTGGGCACCCGCACCTCTGGGAACTG
oligoTJ170	RhoA Y34W fw	CAGTTCCCAGAGGTGTGGGTGCCCACAGTG
oligoTJ171	RhoA Y34W rv	CACTGTGGGCACCCACACCTCTGGGAACTG
oligoTJ72	Rac1 Y32F fw	GCATTTCCTGGAGAATTTATCCCTACTGTC
oligoTJ73	Rac1 Y32F rv	GACAGTAGGGATAAATTCTCCAGGAAATGC
oligoTJ74	Cdc42 Y32F fw	CAAATTTCCATCGGAATTTGTACCGACTG
oligoTJ75	Cdc42 Y32F rv	CAGTCGGTACAAATTCCGATGGAAATTTG
oligoTJ112	NxN Afp18 fw	GGCGGTATATATAAAAATATTAATGATGTACAAGTGAAGGC
oligoTJ113	NXN Afp18 rv	GCCTTCACTTGTACATCATTAATATTTTTATATATACCGCC

Supplementary Table 2. Accession numbers and species of proteins used in this study

Name	*Species*	*Accession number*	*Sequence*
Afp18	*Yersinia ruckeri*	C4ULG3	
TcdB	*Clostridium difficile*	P18177	
RhoA	*Homo sapiens*	P61586	
Rac1	*H. sapiens*	P63000	
Cdc42	*H. sapiens*	P60953	
OGA, O-GlcNAcase	*H. sapiens*	O60502	
OGT, O-glycosyltransferase	*H. sapiens*	O15294	amino acids 322-1041
TAB1	*H. sapiens*	Q15750	amino acids 7-402
ROCK-II	*H. sapiens*	O75116	
PAK	*H. sapiens*	Q13153	amino acids 56-272
Rhotekin	*Mus musculus*	Q8C6B2	amino acids 1-90
CNF1	*Escherichia coli*	Q47106	
Leukemia-associated RhoGEF (LARG)	*H. sapiens*	Q9NZN5	amino acids 766-1138
PDZ-RhoGEF	*H. sapiens*	O15085	amino acids 712-1081
p50RhoGAP	*H. sapiens*	Q07960	
RhoB	*Rattus norvegicus*	P62747	
RhoC	*H. sapiens*	P08134	
Rac2	*H. sapiens*	P15153	
Rac3	*H. sapiens*	P60763	
Cdc42	*H. sapiens*	P60953	
TC10	*H. sapiens*	P17081	
Rheb	*H. sapiens*	Q15382	
Rnd1	*H. sapiens*	Q92730	
Rnd2	*H. sapiens*	P52198	
hRas	*Rattus norvegicus*	P20171	
nRas	*H. sapiens*	P01111	
kRas	*H. sapiens*	P01116	
rRas	*H. sapiens*	P10301	
RalA	*H. sapiens*	P11233	
RalB	*H. sapiens*	P11234	
Rap1B	*H. sapiens*	P61224	
Rap2A	*H. sapiens*	P10114	
Rab5A	*Bos taurus*	Q0IIG7	
CpNagJ	*C. perfringens*	Q0TR53	amino acids 31-618
